# Comprehensive profiling of lncRNAs and mRNAs enriched in small extracellular vesicles for early noninvasive detection of colorectal cancer: diagnostic panel assembly and extensive validation

**DOI:** 10.1002/1878-0261.70086

**Published:** 2025-07-10

**Authors:** Petra Vychytilova‐Faltejskova, Marketa Pavlikova, Lucie Pifkova, Robin Jugas, Tana Machackova, Lenka Radova, Milana Sachlova, Renata Bartosova, Tetiana Samoilenko, Zuzana Feckova, Jana Orlickova, Erika Slamova, Elleni Ponechal Michu, Dagmar Al Tukmachi, Michaela Ruckova, Martina Vodinska, Jan Kotoucek, Tina Catela Ivkovic, Marie Boudna, Lucia Bohovicova, Teodor Stanek, Jana Halamkova, Martin Svoboda, Vladimir Prochazka, Tomas Grolich, Zdenek Kala, Ondrej Slaby

**Affiliations:** ^1^ Centre for Molecular Medicine, Central European Institute of Technology Masaryk University Brno Czech Republic; ^2^ Department of Biology, Faculty of Medicine Masaryk University Brno Czech Republic; ^3^ Department of Gastroenterology and Digestive Endoscopy Masaryk Memorial Cancer Institute Brno Czech Republic; ^4^ Department of Pharmacology and Toxicology Veterinary Research Institute Brno Czech Republic; ^5^ Department of Comprehensive Cancer Care Faculty of Medicine, Masaryk Memorial Cancer Institute Brno Czech Republic; ^6^ Department of Surgical Oncology Faculty of Medicine, Masaryk Memorial Cancer Institute Brno Czech Republic; ^7^ Department of Surgery, Faculty of Medicine, University Hospital Brno Masaryk University Brno Czech Republic

**Keywords:** colorectal cancer, diagnostic panel, high‐throughput expression profiling, long noncoding RNA, small extracellular vesicle

## Abstract

Early diagnosis of colorectal cancer (CRC) is crucial for successful treatment and mortality reduction. In this regard, blood‐based tests play an indispensable role. Current research is focused on molecules actively secreted by tumor cells into small extracellular vesicles (EVs). This four‐phase study included 613 CRC patients, 446 controls, and 120 precancerous lesions. High‐throughput transcriptome profiling of small EVs was performed on samples from 100 CRC patients and 50 controls, followed by extensive validation using reverse transcription quantitative polymerase chain reaction. Diagnostic panels were developed via logistic regression and further characterized by enrolling samples from gastric cancer patients, CRC patients before/after surgery, and samples of tumor tissues/adjacent mucosa. We identified 17 molecules significantly elevated in CRC, with the highest levels in metastatic cases. Additionally, seven of them differentiated controls from precancerous lesions. Two diagnostic panels were developed, enabling early CRC detection with high sensitivity and specificity, outperforming the fecal occult blood test. Furthermore, six molecules were differentially expressed between tumor tissue and mucosa, while seven EV‐enriched molecules decreased significantly after surgery. These findings highlight EVs as key reservoirs of CRC‐associated molecules and a promising source of biomarkers.

## Graphical abstract

Small extracellular vesicles are a promising source of diagnostic molecules. We conducted a comprehensive study, including transcriptome profiling and RT‐qPCR validation on large cohorts of samples. Diagnostic panels enabling sensitive detection of colorectal cancer and precancerous lesions were established. Some molecules were differentially expressed between tumor tissue and adjacent mucosa, decreasing postsurgery, indicating their release into the circulation by tumor cells.

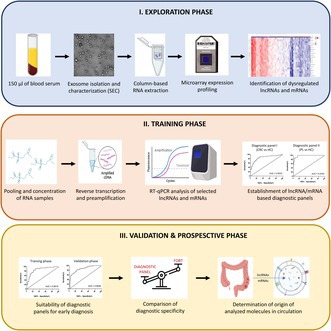

**Abbreviations**
AUC: area under the curve
CD45: protein tyrosine phosphatase C
CMS4: mesenchymal, stromal rich tumor profile
CRC: colorectal cancer
CSRP1: cysteine and glycine‐rich protein 1
DLS: dynamic light scattering
EGR1: early growth response factor 1
EVs: extracellular vesicles
FLOT2: flotillin‐2
FOBT: fecal occult blood test
GC: gastric cancer
HCs: healthy controls
IDT: Integrated DNA Technologies
ITM2B: integral membrane protein 2B
lncRNA: long noncoding RNA
MMCI: Masaryk Memorial Cancer Institute
NAMPT: nicotinamide phosphoribosyl‐transferase
PLs: precancerous lesions
RGS2: regulator of G‐protein signaling 2
ROC: receiver operating characteristic
TEM: transmission electron microscopy
WB: western blot

## 1 Introduction

Colorectal cancer (CRC) is the third most common cancer in the world and the second leading cause of cancer‐related death [1]. Despite the improvements in clinical management and the rapid development of novel therapeutics, the 5‐year survival rate of patients with advanced disease remains still low [2]. Thus, the early diagnosis of CRC is one of the main requirements of successful treatment and a reduction in mortality from this disease [3]. Currently, fecal occult blood test (FOBT) and colonoscopy are considered effective methods for CRC detection and removal of premalignant lesions. Nevertheless, these methods have various limitations including the insufficient sensitivity and reproducibility of FOBT, low adherence rates as well as invasiveness, high cost, lack of accessibility, or extensive patient preparation in case of colonoscopy [4]. Therefore, reliable and minimally invasive biomarkers allowing for early disease detection are urgently needed.

In recent years, researchers have investigated the capabilities of circulating tumor cells as well as different molecules including proteins, lipids, or nucleic acids to serve as novel blood‐based biomarkers [5]. Long noncoding RNAs (lncRNAs) are the widely studied molecules [6]. They are developmentally and tissue‐specific [7] and are associated with a spectrum of biological processes including alternative splicing, modulation of protein activity, alteration of protein localization, and epigenetic regulation [8]. Thus, dysregulation of lncRNAs seems to be an important feature of many complex human diseases, including cancer [9, 10]. Importantly, an increasing number of studies have analyzed the expression levels of lncRNAs in different body fluids [11]. Circulating lncRNAs are associated with lipoprotein complexes or different RNA‐binding proteins [12]. However, a growing body of evidence suggests that they are actively secreted from tumor cells into the circulation, especially via small EVs [13]. Small EVs are 30–150‐nm sized membranous vesicles that are endogenously produced and can participate in intercellular communication by delivering proteins or RNAs to recipient cells [14]. Furthermore, they play critical roles in causing dysregulated local and systemic cellular communication in the tumor microenvironment [15]. An attempt to profile the genetic material enclosed within small EVs demonstrated that lncRNAs account for 3.36% of the total RNA content [16]. Dong et al. [17] investigated the distribution of selected lncRNAs in different serum vesicles. In total, they detected 24 CRC‐related serum lncRNAs, whose expression was greater in small EVs with a size between 45 and 205 nm compared with larger particles. Moreover, increased levels of the lncRNA CRNDE‐h in small EVs can be used to differentiate CRC patients from healthy donors with high sensitivity and specificity, and high expression of this lncRNA was correlated with advanced disease [18]. Expression profiling of lncRNAs present in small EVs via microarrays revealed elevated levels of FOXD2‐AS1, NRIR, and XLOC_009459 in CRC patients compared with healthy donors, and their combination resulted in an area under the curve (AUC) > 0.75 for early‐stage disease [19]. Recently, significantly increased levels of nicotinamide phosphoribosyl‐transferase (NAMPT) and NAMPT‐AS at mRNA and protein expression levels were detected in serum, small EVs, and tumor tissues of CRC patients and associated with poor survival, suggesting a possible involvement of these molecules in CRC pathogenesis [20].

These data indicate that lncRNAs enriched in small EVs could be promising candidates for new diagnostic biomarkers in CRC. However, the main limitations of the earlier studies were the small number of patients and controls in the studied cohorts, the lack of samples from patients with precancerous lesions (PLs), and usually the absence of global expression profiling via a high‐throughput approach, as most of the studied molecules were selected on the basis of the literature and analyzed using RT‐qPCR. Our project aims to overcome these limitations and includes a comprehensive series of experiments with the aim of establishing a diagnostic panel of small EVs‐associated lncRNAs/mRNAs and comprehensively characterizing its analytic parameters. To our knowledge, similar studies with analogous extents have not been published.

## 2 Materials and methods

### 2.1 Patient cohorts and study design

**Fig. 1:**
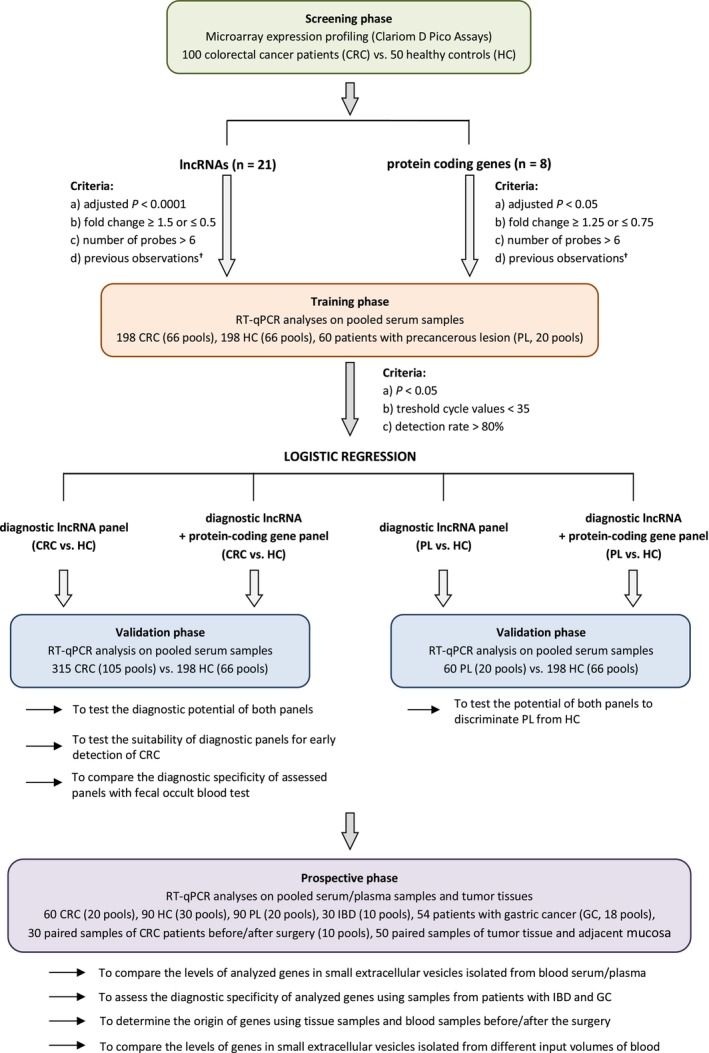
Study design. ^†^Previous observations—combination of the words “lncRNA‐X/mRNA‐Y” and “cancer” and/or combination of the words “lncRNA‐X/mRNA‐Y” and “colorectal cancer,” and/or combination of the words “lncRNA‐X/mRNA‐Y” and “small extracellular vesicles,” and/or combination of the words “lncRNA‐X/mRNA‐Y” and “exosomes” in PubMed; lncRNA—long noncoding RNA, RT‐qPCR” reverse transcriptase quantitative polymerase chain reaction.

This study was designed as a 4‐phase biomarker study (Fig. 1). In total, 613 patients with histopathologically verified CRC who underwent resection from 2006 through 2024 at Masaryk Memorial Cancer Institute (MMCI, Brno, Czech Republic) or Faculty Hospital Brno (Czech Republic), 446 serum samples from healthy controls (HCs) collected at the Cancer Prevention Center between 2021 and 2024, and 120 samples from patients with different types of PLs (MMCI) were included in the study and divided into screening, training, and validation cohorts (Table S1). In addition, 54 samples from patients with gastric cancer (MMCI; Table S2) and 30 paired samples from CRC patients before and 3 months after surgery (average age 64 years, 21 males, 9 females; Faculty Hospital Brno Bohunice) were analyzed. Finally, 50 pairs of tumoral and adjacent nontumoral tissues were used in the study (MMCI, Table S3). Fresh tissues were obtained after surgical resection and immediately placed in liquid nitrogen. All blood samples were collected prior to surgery, and CRC patients did not receive any neoadjuvant treatment. HCs had no prior diagnosis of any malignancy. Written informed consent was obtained from all participants, and the study was approved by the local Ethical Board at MMCI (2019/1842/MOU) and by the Research Ethics Committee at Masaryk University (EKV‐2019‐053).

### 2.2 Blood collection procedure and serum isolation

The blood was collected using S‐Monovette® Serum CAT, 4‐mL collection tubes and 21 G butterfly needle (Sarstedt, Nümbrecht, Germany) to avoid platelet activation and hemolysis. Subsequently, the samples were placed in an upright position and allowed to coagulate for 45 min at room temperature. Then, the samples were immediately spun down at 1500 × ***g***, 20 min at 15 °C using a swing‐out rotor without applying a break to ensure a clean serum supernatant and to avoid cold‐induced platelet activation and EV contamination [21]. Next, the serum was collected while leaving approximately 0.5 cm safety margin on top of the clot, transferred to a new RNase‐free tubes and stored at −80 °C before further purification and isolation of small EVs.

### 2.3 Isolation and characterization of small extracellular vesicles

Small EVs were isolated from 150 μL·500μL^−1^ of serum using qEVsingle/35 nm or qEVoriginal/35 nm columns, respectively (iZON Science Ltd., Oxford, UK), according to our optimized procedure [22]. All the samples were stored at −80 °C before RNA isolation. The quality and quantity of isolated small EVs were assessed by dynamic light scattering (DLS), transmission electron microscopy (TEM), and western blot (WB) analysis as described previously [22] according to ISEV recommendations [21]. A list of antibodies is provided in Table S4.

### 2.4 Isolation of RNA from small extracellular vesicles and from tissue

Total RNA was isolated using the Monarch® Total RNA Miniprep Kit (New England Biolabs, Ipswich, USA) according to the manufacturer's recommendations, treated with DNase I, and eluted with 50 μL of DEPC‐treated water. The concentration of RNA was too low to be reliably determined either by Qubit or Bioanalyzer. All analyzed tissues were homogenized (Retch MM301), and total RNA was isolated using the *mir*Vana miRNA Isolation Kit (Invitrogen, Waltham, MA, USA). The concentration and purity of RNA isolated from tissues were determined spectrophotometrically by measuring its optical density using a Nanodrop ND‐1000 (Thermo Fisher Scientific, Waltham, MA, USA). The isolated RNA was stored at −80 °C before the RT‐qPCR analysis.

### 2.5 Microarray analysis

Microarray analysis was performed via the Clariom D Pico Assay, human (Applied Biosystems, Waltham, MA, USA), according to the manufacturer's recommendations. The pre‐IVT amplification was performed in a thermal cycler for 2 min at 95 °C, 12 cycles of 30 s at 94 °C and 5 min at 70 °C, and then for at least 2 min at 4 °C. The arrays were stained using an Affymetrix GeneChip Fluidics Station 450 according to the FS450_0001 protocol and scanned with an Affymetrix GeneChip n 3000 7G (Santa Clara, CA, USA).

### 2.6 Statistical analysis of microarray data

A quality control check of the microarray data was performed using the “arrayQualityMetrics.” Chip definition files were created using probe sequences from BRAINARRAY CDF [23] and the GENCODE (v40) database as a target as described previously [22]. The 25‐bp‐long probe sequences were aligned using BLASTn. Promiscuous probes mapping to multiple different genes were removed according to OPOG principle (one probe–one gene). A previously published script [24] was applied to build our own custom CDF and the “pdInfoBuilder” was used to create the R package. Differential expression analysis was performed with the “oligo” and “limma” packages. The expression values were normalized by RMA algorithm and *P*‐values were adjusted by Benjamini‐Hochberg procedure.

### 2.7 Reverse transcription and RT‐qPCR

RNA samples were pooled in groups of three according to sex, diagnosis, and clinical stage and concentrated to 6 μL using a Concentrator Plus 5305 Vacuum Centrifuge (Eppendorf AG, Hamburg, Germany). Complementary DNA was synthesized using 5 μL of total RNA and the High‐Capacity cDNA Reverse Transcription Kit according to the manufacturer's recommendations. A preamplification step was performed by mixing 2.5 μL of cDNA with 5 μL of TaqMan PreAmp Master Mix (all Applied Biosystems) and 2.5 μL of pooled 200 nm primers synthesized by Integrated DNA Technologies (IDT) (Table S5) at 95 °C for 10 min, 95 °C for 15 s, and 60 °C for 4 min (14 cycles), 99 °C for 10 min, and holding at 4 °C. Quantitative PCR was carried out mixing 5 μL of PowerUP SYBR Green Master Mix (Applied Biosystems), 2.5 μL of preamplified cDNA diluted 20× in 1× TE buffer, 0.5 μL of nuclease‐free water, and 2 μL of 1 μm IDT primers. In the case of tissue samples, cDNA was synthesized using 2 μg of total RNA and the High‐Capacity cDNA Reverse Transcription Kit (Applied Biosystems) according to the manufacturer's recommendations. Quantitative PCR was carried out using 1 μL of cDNA, 2 μL of nuclease‐free water, 2 μL of 1 μm IDT primers, and 5 μL of PowerUP SYBR Green Master Mix. Fluorescence was measured with a QuantStudio 12 K Flex Real‐Time PCR system (all Applied Biosystems).

### 2.8 Statistical analysis of RT‐qPCR data

The threshold cycle data were calculated by QuantStudio 12 K Flex software. The average expression levels of all genes were normalized via the combination of GAPDH (Hs99999905_m1) and ACTB (Hs99999903_m1) and subsequently analyzed by the $2^{-\Delta C_{t}}$ method. These reference genes were selected using the GenEx software. The expression in tissues was normalized using PMM1 (Hs00160195_m1), which was previously validated [25]. Statistical differences between the levels of analyzed genes in the serum samples of CRC patients and HCs were evaluated by a two‐tailed nonparametric Mann–Whitney *U*‐test. Paired samples before and after surgery and tissue samples were analyzed using a two‐tailed nonparametric Wilcoxon test for paired samples. The Kruskal–Wallis test was used to analyze the correlation between gene expression levels and clinicopathological features. To generate a diagnosis‐related signature, the top 13 lncRNAs and 5 mRNAs meeting the established criteria were introduced into a bidirectional stepwise logistic regression model on the training cohort. The final model was taken as the one that minimizes the Akaike information criterion. The selection was made within R‐freeware (R‐4.4.1) by the “step” function. A receiver operating characteristic (ROC) curve was generated for further analysis of the DXscore and calculation of sensitivity and specificity. The maximum Youden index was used to obtain the optimal cutoff values. All calculations were performed using GraphPad Prism version 5.00 (GraphPad Software, La Jolla, CA, USA) and the *R* environment (R‐4.4.1; R Development Core Team, Vienna, Austria). *P*‐values of less than 0.05 were considered statistically significant.

## 3 Results

### 3.1 Isolation and characterization of small EVs

**Fig. 2:**
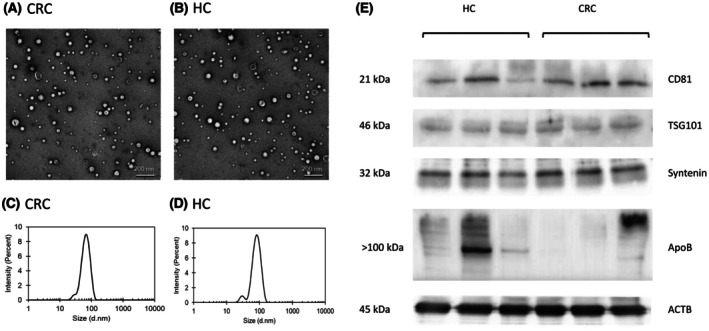
Characterization of small extracellular vesicles. (A, B) transmission electron microscopy (scale bar = 200 nm), (C, D) dynamic light scattering, and (E) western blot analysis (*n*_CRC_ = 3, *n*_HC_ = 3; individual proteins were detected on different gels or different parts of a gel). CRC—Representative samples of colorectal cancer, HC—representative samples of healthy controls, ApoB—Apolipoprotein B, ACTB—β‐Actin.

In total, 1293 samples were involved in the current research. Thus, the detailed characterization of small EVs was performed only on a subset of 20 CRC patients and 20 HCs. The mean particle size for CRC patient samples analyzed was 92 ± 4 nm, while that of HCs was 84 ± 7 nm. The polydispersity index was determined to be 0.219 ± 0.064 in CRC patients and 0.210 ± 0.047 in case of HCs. The concentration of particles was 2.1 · 10^13^ ± 1.7 · 10^13^ mL^−1^ in CRC patients and 7.2 · 10^12^ ± 5.3 · 10^11^ mL^−1^ in HCs. TEM analysis confirmed the presence of rounded, cup‐shaped vesicles corresponding to the size of the small EVs. WB analysis revealed the expression of characteristic markers in the isolated vesicles. In addition, lipoprotein contamination was assessed by analyzing the expression of ApoB protein. While only a weak signal was detected in samples from CRC patients, the signal intensity varied widely among the samples from HCs. Overall, the results confirmed the presence of vesicles of appropriate size, shape, and sufficient purity in the isolated samples, although the lipoproteins were coisolated to some extent (Fig. 2, Fig. S1).

### 3.2 Microarray expression profiling of serum‐derived small EVs

**Fig. 3:**
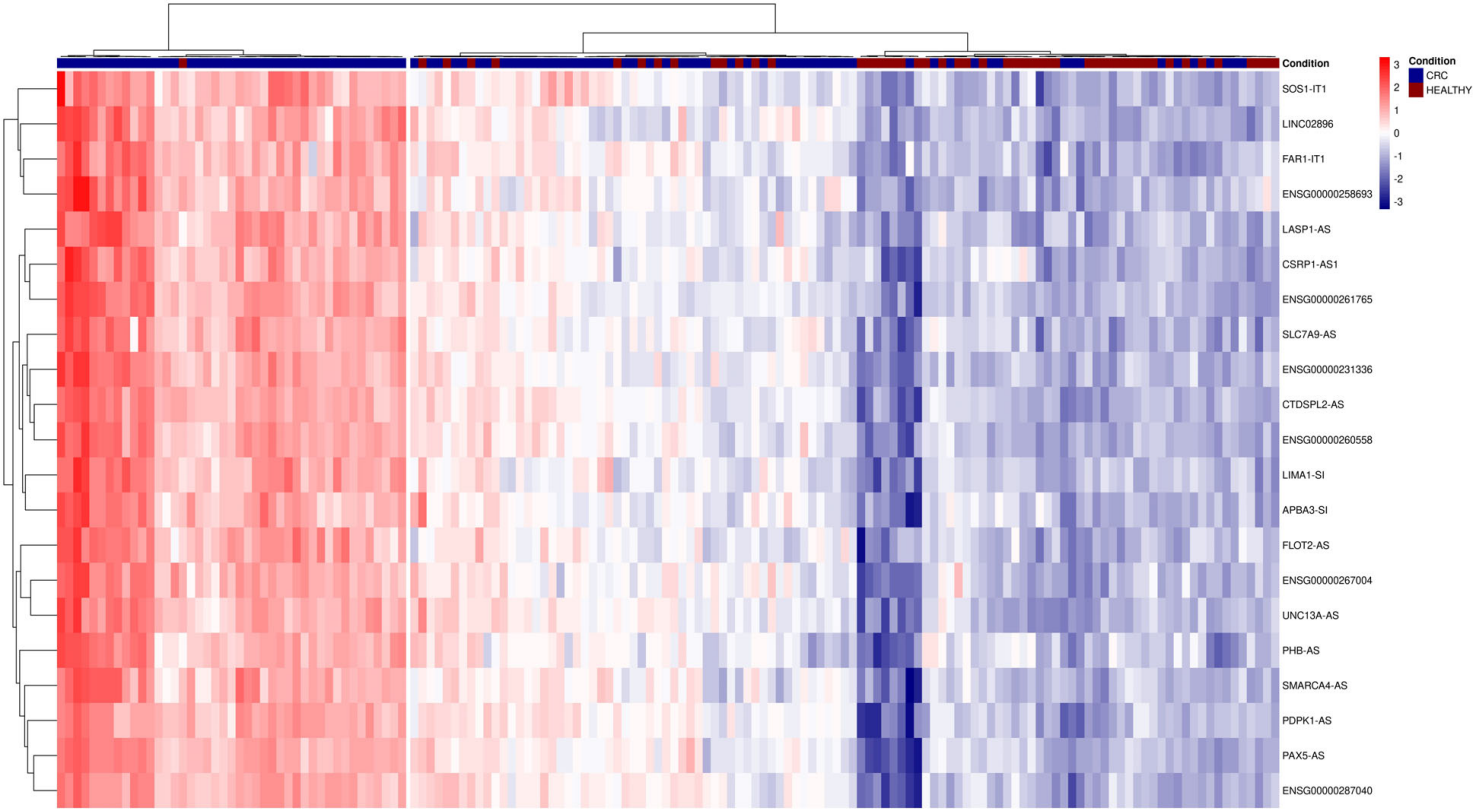
Microarray expression profiling of serum‐derived small extracellular vesicles. Hierarchical clustering of 100 colorectal cancer (CRC) samples and 50 healthy control samples on the basis of the significantly different expression (adjusted *P* < 0.0001) of 21 long noncoding RNAs enriched in serum small extracellular vesicles.

In total, 4014 transcripts with different levels between CRC patients and HCs were identified (adjusted *P* < 0.05; Table S6). Among these genes, 1469 were assigned to lncRNAs (36.6%), whereas protein‐coding genes represented the second most common gene biotype (1166 transcripts; 29%; Table 1). Hierarchical clustering proved that it is possible to distinguish CRC samples from HCs using the levels of mRNAs and lncRNAs, separately or in combination (Fig. S2). Subsequently, more stringent criteria were set (Fig. 1) to identify lncRNAs/mRNAs with the highest potential to discriminate the two groups of samples. In total, we obtained 21 lncRNAs (Fig. 3, Table 2A) and 8 mRNAs (Table 2B) that met our requirements and were chosen for further evaluation.

**Table 1:** Overview of the significantly dysregulated gene biotypes. Significantly dysregulated gene biotypes in the serum‐derived small extracellular vesicles of colorectal cancer patients (*n* = 100) compared with those of healthy controls (*n* = 50) analyzed by Clariom D Pico Assays (adjusted *P* < 0.05).

Gene biotype	Class of RNA	Total number of transcripts	Percentage from all identified genes [%]	Percentage from particular gene biotype [%]
**Noncoding RNAs**		**1893**	**47.2%**	—
	lncRNAs	1469	36.6%	77.6%
	miscRNA	209	5.2%	11.0%
	miRNA	122	3.0%	6.4%
	snRNA	63	1.6%	3.3%
	snoRNA	26	0.7%	1.4%
	rRNA	3	0.1%	0.2%
	scaRNA	1	0.0%	0.1%
**Protein‐coding**		**1166**	**29.0%**	—
**Pseudogenes**		**891**	**22.2%**	—
	Processed	700	17.5%	78.5%
	Unprocessed	144	3.6%	16.2%
	rRNA	33	0.8%	3.7%
	Unitary	13	0.3%	1.5%
	Polymorphic	1	0.0%	0.1%
**Others**		**64**	**1.6%**	—

**Table 2:** The list of 21 long noncoding RNAs and 8 mRNAs with different levels between serum‐derived small extracellular vesicles of colorectal cancer patients (*n* = 100) and healthy controls (*n* = 50) in the exploration phase of the study.

Ensembl id	Gene name	Fold change	Level	*P*	Adj *P*
**(A) Long noncoding RNAs**					
ENSG00000259215	LINC02896	1.51	5.99	1.31 · 10^−14^	7.51 · 10^−10^
ENSG00000229692	SOS1‐IT1	1.70	5.84	2.16 · 10^−13^	3.10 · 10^−09^
ENSG00000263466	LASP1‐AS	1.70	5.29	3.54 · 10^−13^	4.07 · 10^−09^
ENSG00000259563	CTDSPL2‐AS	1.81	7.51	1.79 · 10^−12^	1.41 · 10^−08^
ENSG00000260558	ENSG00000260558	1.68	7.94	2.22 · 10^−12^	1.41 · 10^−08^
ENSG00000258885	PAX5‐AS	1.88	8.42	3.69 · 10^−12^	1.93 · 10^−08^
ENSG00000267555	SLC7A9‐AS	1.59	7.35	4.69 · 10^−12^	2.24 · 10^−08^
ENSG00000257298	LIMA1‐SI	1.92	5.94	6.47 · 10^−12^	2.34 · 10^−08^
ENSG00000261140	PDPK1‐AS	1.61	8.17	6.93 · 10^−12^	2.34 · 10^−08^
ENSG00000266936	SMARCA4‐AS	1.84	6.45	1.21 · 10^−11^	3.46 · 10^−08^
ENSG00000264304	FLOT2‐AS	1.80	4.55	1.40 · 10^−11^	3.65 · 10^−08^
ENSG00000254791	FAR1‐IT1	1.50	5.77	1.60 · 10^−11^	3.84 · 10^−08^
ENSG00000224536	CSRP1‐AS1	1.54	6.55	1.76 · 10^−11^	4.03 · 10^−08^
ENSG00000261765	ENSG00000261765	1.64	7.77	7.08 · 10^−11^	1.20 · 10^−07^
ENSG00000287040	RP11‐190A12	1.83	8.70	1.36 · 10^−10^	2.04 · 10^−07^
ENSG00000258693	ENSG00000258693	1.87	5.18	6.32 · 10^−10^	6.05 · 10^−07^
ENSG00000231336	RP11‐110G2	1.69	6.60	1.12 · 10^−09^	8.58 · 10^−07^
ENSG00000269752	UNC13A‐AS	1.76	6.04	1.43 · 10^−09^	1.01 · 10^−06^
ENSG00000267004	ENSG00000267004	1.79	7.43	7.29 · 10^−09^	3.27 · 10^−06^
ENSG00000267138	APBA3‐SI	1.76	6.56	7.47 · 10^−09^	3.33 · 10^−06^
ENSG00000262039	PHB‐AS	2.58	5.98	7.61 · 10^−08^	2.02 · 10^−05^
**(B) mRNAs**					
ENSG00000213402	PTPRCAP	0.67	3.86	4.23 · 10^−05^	2.59 · 10^−03^
ENSG00000253506	NACA2	0.75	3.36	4.66 · 10^−05^	2.76 · 10^−03^
ENSG00000120738	EGR1	1.36	5.24	1.27 · 10^−04^	5.80 · 10^−03^
ENSG00000117091	CD48	0.74	4.82	3.35 · 10^−04^	1.13 · 10^−02^
ENSG00000136156	ITM2B	1.36	5.35	6.70 · 10^−04^	1.77 · 10^−02^
ENSG00000197061	H4C3	0.53	8.33	1.02 · 10^−03^	2.32 · 10^−02^
ENSG00000116741	RGS2	1.34	4.59	1.55 · 10^−03^	3.02 · 10^−02^
ENSG00000121966	CXCR4	1.36	3.66	3.43 · 10^−03^	4.89 · 10^−02^

### 3.3 Training phase of the study

During the primer design process, one of the lncRNAs (ENSG00000267004) had to be discarded because its sequence was too short and formed secondary structures and nonspecific products. The expression of the remaining 28 genes was successfully analyzed in 66 pools of CRC samples, 66 pools of HCs, and 20 pools of PLs using RT‐qPCR. As summarized in Table 3, the levels of all lncRNAs were elevated in CRC patients compared with HCs. However, only 13 of them were confirmed to be significantly dysregulated between these two groups of samples (*P* < 0.05; Fig. S3). Similarly, the levels of five of the analyzed mRNAs were significantly increased in CRC patients (*P* < 0.05; Fig. S4A), which is in accordance with the results of expression profiling with the exception of PTPRCAP. Concerning the PLs, SOS1‐IT1, RGS2, and EGR1 allowed us to differentiate between those samples and HCs, while the levels of 11 lncRNAs and three mRNAs were significantly increased in CRC patients compared to PLs (Figs S5 and S6A–C). The diagnostic performance of individual candidate biomarkers is summarized in Tables S7 and S8. Furthermore, the levels of lncRNAs as well as EGR1 and CXCR4 were greater in patients with metastatic disease than in patients with localized disease. However, only CSRP1‐AS varied significantly among particular clinical stages (*P* = 0.0211) and its levels increased progressively from stage I to stage IV (Fig. S6D–F). Concerning the clinical diagnosis, there were no significant differences in lncRNA levels between patients with C18 and those with C19. However, significantly increased levels of LIMA1‐SI (*P* = 0.0451), CTDSPL2AS (*P* = 0.0184), SMARCA4‐AS (*P* = 0.0180), CSRP1‐AS1 (*P* = 0.0090), PAX5‐AS (*P* = 0.0086), RP11‐110G2 (*P* = 0.0451), UNC13A‐AS (*P* = 0.0297), RP11‐190A12 (*P* = 0.0277), ENSG00000260558 (*P* = 0.0119), EGR1 (*P* = 0.0014), RGS2 (*P* = 0.0046), CXCR4 (*P* = 0.0014), ITM2B (*P* = 0.0101), and PTPRCAP (*P* = 0.0019) were found in samples from patients with colon cancer (C18 + C19) compared to the samples from patients with rectal cancer (C20). No significant changes were found neither between the samples from men and women, nor among the samples with different tumor grades.

**Table 3:** Median levels of analyzed molecules in the training phase of the study. Levels of long noncoding RNAs and mRNAs in small extracellular vesicles isolated from the blood serum of colorectal cancer (CRC) patients and healthy controls (HC). FC – fold change.

Gene biotype	Gene name	Median FC (CRC vs. HC)	Median CRC levels (min–max)	Median HC levels (min–max)	*P*
lncRNA	**SOS1‐IT1**	3.40	2.21 (0.00–101.60)	0.65 (0.00–9.34)	**< 0.0001**
lncRNA	**RP11‐110G2**	2.66	3.35 (0.00–137.50)	1.26 (0.00–26.69)	**< 0.0001**
lncRNA	**CSRP1‐AS1**	2.44	2.57 (0.00–234.30)	1.05 (0.00–16.19)	**< 0.0001**
lncRNA	**PDPK1‐AS**	3.41	5.04 (0.00–153.00)	1.48 (0.00–13.83)	**0.0005**
lncRNA	**SMARCA4‐AS**	2.28	2.74 (0.00–100.70)	1.20 (0.00–110.40)	**0.0007**
lncRNA	**FAR1‐IT1**	2.55	1.60 (0.00–149.90)	0.63 (0.00–25.41)	**0.0013**
lncRNA	**ENSG00000261765**	2.34	5.97 (0.37–126.50)	2.55 (0.00–41.06)	**0.0025**
lncRNA	**SLC7A9‐AS**	1.89	2.54 (0.00–116.90)	1.34 (0.00–33.90)	**0.0040**
lncRNA	**PAX5‐AS**	2.60	0.39 (0.00–16.96)	0.15 (0.00–13.06)	**0.0105**
lncRNA	**PHB‐AS**	1.84	1.98 (0.00–70.04)	1.08 (0.00–28.07)	**0.0136**
lncRNA	**RP11‐190A12**	2.52	2.25 (0.00–80.47)	0.89 (0.00–27.31)	**0.0141**
lncRNA	**FLOT2‐AS**	1.86	2.40 (0.00–62.05)	1.29 (0.00–26.76)	**0.0214**
lncRNA	**UNC13A‐AS**	2.41	1.48 (0.00–94.13)	0.62 (0.00–32.11)	**0.0241**
lncRNA	ENSG00000260558	2.20	1.62 (0.00–64.35)	0.73 (0.00–25.94)	0.0624
lncRNA	LIMA1‐SI	1.51	1.02 (0.00–37.07)	0.67 (0.00–263.80)	0.0741
lncRNA	LASP1‐AS	1.86	1.28 (0.00–41.94)	0.68 (0.00–21.94)	0.0786
lncRNA	APBA3‐SI	1.42	1.72 (0.00–60.29)	1.21 (0.00–154.60)	0.4405
lncRNA	CTDSPL2‐AS	1.41	1.68 (0.00–60.91)	1.19 (0.00–154.80)	0.6638
lncRNA	LINC02896	1.24	1.61 (0.00–56.15)	1.30 (0.00–122.90)	0.6671
lncRNA	ENSG00000258693	1.37	1.54 (0.00–49.37)	1.12 (0.00–50.18)	0.7311
mRNA	**EGR1**	3.29	8.95 (1.39–127.70)	2.72 (0.25–33.98)	**< 0.0001**
mRNA	**RGS2**	2.18	13.05 (2.02–140.50)	5.98 (0.02–118.60)	**< 0.0001**
mRNA	**CXCR4**	2.11	4.22 (0.55–94.04)	2.00 (0.14–37.87)	**< 0.0001**
mRNA	**ITM2B**	2.15	15.50 (2.57–100.10)	7.20 (0.18–91.36)	**0.0001**
mRNA	**PTPRCAP**	1.43	5.17 (0.65–85.72)	3.62 (0.06–25.26)	**0.0079**
mRNA	CD48	1.13	4.20 (0.53–104.90)	3.73 (0.14–24.17)	0.2920
mRNA	NACA2	1.28	1.05 (0.03–26.58)	0.82 (0.03–15.25)	0.4258
mRNA	H4C3	0.92	8.66 (0.02–97.12)	9.44 (0.03–210.6)	0.6403

Bold values indicate statistical significance (*P* < 0.05).

Subsequently, 13 lncRNAs, whose levels were different between CRC patients and HCs, were used for a bidirectional stepwise logistic regression model, and the diagnostic score was calculated according to the following formula: DXscore = −0.384 – 0.123 · PHB‐AS + 0.0789 · CSRP1‐AS1 + 0.108 · SOS1‐IT1 + 0.0658 · PDPK1‐AS + 0.182 · RP11‐110G2–0.165 · SLC7A9‐AS – 0.0538 · ENSG00000261765. ROC analyses proved that this 7‐lncRNA‐based panel enables distinguishing samples of CRC patients from HCs with a sensitivity of 76% and specificity of 71%, with an AUC = 0.8104 (Fig. S7A, Table S9A). Similarly, a 3‐lncRNA‐based panel was assessed for its ability to differentiate PLs from HC samples (DXscore = 0.628 + 0.089 · PHB‐AS +1.052 · SOS1‐IT1–2.407 · LASP1‐AS) with a sensitivity of 90% and specificity of 85%, with an AUC = 0.9220 (Fig. S7B, Table S10A).

**Fig. 4:**
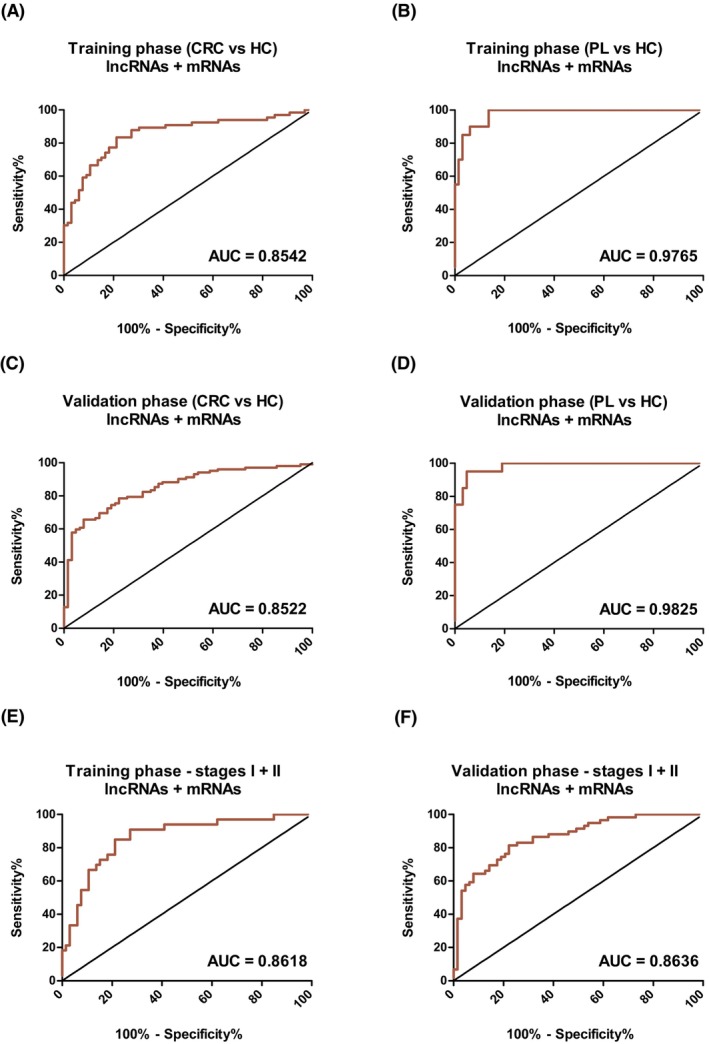
Performance of established diagnostic panels using small extracellular vesicles isolated from serum samples of colorectal cancer patients (CRC), healthy controls (HC), and patients with precancerous lesions (PL). (A) ROC analysis using the expression of 7‐lncRNA‐based panel augmented by mRNA—the training cohort (AUC = 0.8542, *n*_CRC_ = 66, *n*_HC_ = 66). (B) ROC analysis using the expression of 3‐lncRNA‐based panel augmented by mRNAs—the training cohort (AUC = 0.9765, *n*_PL_ = 20, *n*_HC_ = 66). (C) ROC analysis using the expression of 7‐lncRNA‐based panel augmented by mRNA—the validation cohort (AUC = 0.8522, *n*_CRC_ = 105, *n*_HC_ = 66). (D) ROC analysis using the expression of 3‐lncRNA‐based panel augmented by mRNAs—the validation cohort (AUC = 0.9825, *n*_PL_ = 20, *n*_HC_ = 66). (E) ROC analysis using the expression of 7‐lncRNA‐based panel augmented by mRNA—the training cohort, stages I + II (AUC = 0.8618, *n*_CRC_ = 33, *n*_HC_ = 66). (F) ROC analysis using the expression of 7‐lncRNA‐based panel augmented by mRNA—the validation cohort, early stages I + II (AUC = 0.8636, *n*_CRC_ = 59, *n*_HC_ = 66). ROC—receiver operating characteristics, AUC—area under the curve. The red lines correspond to ROC curves.

To further increase the diagnostic performance of the obtained panels, differentially enriched mRNAs were included in a bidirectional stepwise logistic regression model and the diagnostic score was recalculated: DXscore = −2.3943 + 1.2166 · SOS1‐IT1 + 0.4333 · PDPK1‐AS – 0.4597 · SMARCA4‐AS – 0.6323 · FAR1‐IT1 – 0.3388 · ENSG00000261765–0.5385 · SLC7A9‐AS + 0.5669 · EGR1. This improved panel augmented by EGR1 enabled distinguishing samples from CRC patients from HCs with a sensitivity of 83% and specificity of 79%, with an AUC = 0.8542 (Fig. 4A, Table S9B). Additionally, a combined panel of three lncRNAs and three mRNAs was used to differentiate PLs from HCs (DXscore = − 1.67 – 0.33 · PHB‐AS + 2.425 · SOS1‐IT1–1.269 · LASP1‐AS – 0.463 · RGS2–0.404 · EGR1 + 0.276 · ITM2B) with a sensitivity of 100% and specificity of 86%, with an AUC = 0.9765 (Fig. 4B, Table S10B). Since mRNAs contained in small EVs contributed to higher sensitivity and specificity of diagnostic panels, not only lncRNAs (*n* = 14) but also significantly dysregulated mRNAs (*n* = 5) were selected for the validation phase of the study.

### 3.4 Validation phase of the study

During the validation phase, 19 selected genes were successfully analyzed in 105 pools of CRC samples, 66 pools of HCs, and 20 pools of PLs using RT‐qPCR. As summarized in Table 4, significantly different levels of 13 lncRNAs and four mRNAs were confirmed between samples from CRC patients and HCs (*P* < 0.05; Figs S8 and S4B). Concerning the PLs, PHB‐AS, UNC13A‐AS, LASP1‐AS, FLOT2‐AS, RGS2, EGR1, and PTPRCAP enabled to differentiate between HCs and patients with lesions (Table S11A). In addition, the levels of PHB‐AS, SOS1‐IT1, UNC13A‐AS, FLOT2‐AS, RGS2, EGR1, and ITM2B were significantly decreased in samples from PLs compared to those from CRC patients (Table S11B). The diagnostic performance of individual candidate biomarkers is summarized in Tables S7 and S8. Similar to the training phase of the study, the levels of CSRP1‐AS varied significantly among particular clinical stages (*P* = 0.0382), as did the levels of SOS1‐IT1 (*P* = 0.0326), ENSG00000261765 (*P* = 0.0065), and ITM2B (*P* = 0.0130). Similar to the training phase of the study, there were no significant changes related to sex or different tumor grades.

**Table 4:** Median levels of analyzed molecules in the validation phase of the study. Levels of long noncoding RNAs and mRNAs in small extracellular vesicles isolated from the blood serum of colorectal cancer (CRC) patients and healthy controls (HC). FC‐fold change.

Gene biotype	Gene name	Median FC (CRC vs. HC)	Median CRC levels (min–max)	Median HC levels (min–max)	*P*
lncRNA	**FLOT2‐AS**	2.88	18.29 (1.25–258.00)	6.35 (0.90–67.82)	**< 0.0001**
lncRNA	**SOS1‐IT1**	2.76	13.61 (0.50–171.20)	4.93 (0.67–52.80)	**< 0.0001**
lncRNA	**RP11‐110G2**	2.71	22.47 (1.75–247.80)	8.30 (0.99–74.09)	**< 0.0001**
lncRNA	**PHB‐AS**	2.49	19.00 (1.49–259.40)	7.64 (0.91–89.56)	**< 0.0001**
lncRNA	**PDPK1‐AS**	2.34	27.16 (1.29–297.60)	11.59 (1.94–111.3)	**< 0.0001**
lncRNA	**RP11‐190A12**	2.22	15.55 (0.54–165.10)	7.00 (1.54–59.55)	**< 0.0001**
lncRNA	**SMARCA4‐AS**	2.09	16.76 (0.64–237.30)	8.01 (1.01–81.07)	**< 0.0001**
lncRNA	**CSRP1‐AS1**	2.09	17.50 (0.78–226.10)	8.38 (0.92–84.41)	**< 0.0001**
lncRNA	**UNC13A‐AS**	2.05	9.41 (0.46–126.20)	4.59 (0.71–52.35)	**< 0.0001**
lncRNA	**ENSG00000261765**	2.03	24.91 (0.80–155.30)	12.27 (2.22–85.23)	**< 0.0001**
lncRNA	**SLC7A9‐AS**	1.86	12.71 (0.43–155.00)	6.83 (1.15–81.72)	**< 0.0001**
lncRNA	**PAX5‐AS**	1.91	3.46 (0.08–35.09)	1.81 (0.23–25.03)	**0.0001**
lncRNA	**FAR1‐IT1**	1.70	11.80 (0.51–130.30)	6.94 (1.11–76.87)	**0.0003**
mRNA	**EGR1**	2.47	43.22 (0.85–384.40)	17.53 (3.58–165.00)	**< 0.0001**
mRNA	**RGS2**	2.36	47.84 (2.78–317.70)	20.23 (6.85–81.30)	**< 0.0001**
mRNA	**CXCR4**	2.16	25.20 (1.40–265.80)	11.68 (2.16–91.11)	**< 0.0001**
mRNA	**ITM2B**	1.81	31.44 (1.57–237.80)	17.34 (7.89–71.05)	**< 0.0001**

Bold values indicate statistical significance (*P* < 0.05).

The performance of diagnostic panels obtained during the training phase of the study was subsequently assessed using the previously described DXscores. In the case of CRC patients, ROC analyses proved that our 7‐lncRNA‐based panel enables to distinguish those samples from HCs with a sensitivity of 74% and specificity of 70% with an AUC = 0.7879 (Fig. S7C, Table S9A), while the improved panel augmented by EGR1 enabled to distinguish these two cohorts with a sensitivity of 78% and specificity of 78% with AUC = 0.8522 (Fig. 4C, Table S9B). Similarly, in the case of PLs the 3‐lncRNA‐based panel enabled to differentiate lesions from the samples from HCs with a sensitivity of 85% and specificity of 90% with an AUC = 0.9429 (Fig. S7D, Table S10A), whereas the combined panel of three lncRNAs and three mRNAs distinguished these two groups of samples with a sensitivity of 95% and specificity of 95%, with an AUC = 0.9825 (Fig. 4D, Table S10B). These results are consistent with the training phase of the study.

### 3.5 Suitability of diagnostic panels for the early detection of colorectal cancer

The suitability of the obtained panels for the early diagnosis of CRC was tested using only patients' samples with localized disease. The ROC analysis confirmed the high sensitivity and specificity of both panels in the training phase of the study (lncRNAs‐based panel: AUC = 0.8085; lncRNAs/mRNAs‐based panel: AUC = 0.8618) as well as in the independent validation phase (lncRNAs‐based panel: AUC = 0.8004; lncRNAs/mRNAs‐based panel: AUC = 0.8636) as shown in Fig. 4E,F, Fig. S7E,F, and summarized in Table S9. These data confirm the high diagnostic potential of the established panels.

### 3.6 Diagnostic specificity of small EVs‐enriched lncRNAs and mRNAs

First, the diagnostic specificity of the established panels was compared with that of the FOBT. In total, 20 samples from HCs were tested. On the basis of the results of the FOBT, all involved individuals were positive for blood in the stool and thus underwent a colonoscopy, which was negative in all cases. In contrast, the lncRNA‐based panel confirmed the negative findings in 11 of the 20 samples (55%; cut‐off = 0.8835), whereas the panel augmented with EGR1 confirmed the negative findings in 16 of the 20 samples (80%; cut‐off = 6.510). These data indicate a higher specificity of established panels compared with FOBT. Second, the specificity of small EVs‐enriched lncRNAs and mRNAs was analyzed using 18 pools of samples from gastric cancer (GC) patients. None of the analyzed molecules had significantly different levels between GC patients and HCs, except for PAX5‐AS (*P* = 0.0313), SLC7A9‐AS (*P* = 0.0007), and PTPRCAP (*P* = 0.0026), whose levels were significantly lower in GC patients, which is exactly the opposite result to that observed in CRC patients.

### 3.7 Origin of diagnostic small EVs‐enriched lncRNAs and mRNAs in circulation

**Fig. 5:**
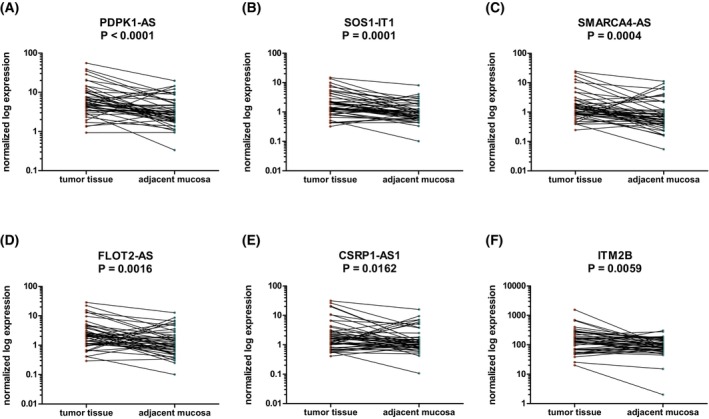
Expression levels of selected RNAs in tissues. Significantly dysregulated long noncoding RNAs and mRNAs between 50 paired samples of tumor tissue and the corresponding adjacent mucosa of colorectal cancer patients (two‐tailed nonparametric Wilcoxon test for paired samples, *P* < 0.05). The red and green dots correspond to tumor tissue and adjacent mucosa samples, respectively.

**Fig. 6:**
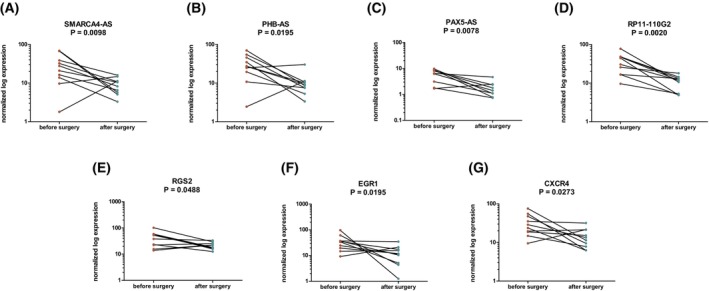
Decreased levels of selected RNAs in small extracellular vesicles of colorectal cancer (CRC) patients after tumor resection. Significantly decreasing levels of long noncoding RNAs and mRNAs in small extracellular vesicles of colorectal cancer patients (*n* = 10) 3 months after tumor removal compared to the levels before the surgery (two‐tailed nonparametric Wilcoxon test for paired samples, *P* < 0.05). The red and green dots correspond to before and after surgery samples, respectively.

To determine whether the identified lncRNAs and mRNAs enriched in small EVs are associated with CRC and might be actively secreted into the circulation by tumor cells, we analyzed their expression in 50 paired samples of tumor tissue and corresponding adjacent mucosa. We detected PDPK1‐AS, SOS1‐IT1, SMARCA4‐AS, FLOT2‐AS, CSRP1‐AS1, and ITM2B to be upregulated in tumor tissue compared with adjacent mucosa (Fig. 5, Table S12). Furthermore, the levels of 13 lncRNAs and 4 mRNAs were assessed in small EVs isolated from 30 paired samples from CRC patients before surgical resection and 3 months after tumor removal. We detected a significant decrease in SMARCA4‐AS, PHB‐AS, PAX5‐AS, RP11‐110G2, RGS2, EGR1, and CXCR4 in samples after surgical resection (Fig. 6).

## 4 Discussion

The prognosis of CRC varies depending on the stage at diagnosis. While the early‐stage CRC is highly treatable, treatment options for advanced stages are limited. In practice, it can be challenging to detect the disease in its early stages as it has very nonspecific symptoms. Thus, the most effective method for preventing CRC and reducing mortality is regular screening, which allows both early detection and removal of preneoplastic lesions. Most screening programs throughout the world incorporate fecal testing including FOBT or fecal immunochemical test as well as colonoscopy or flexible sigmoidoscopy [26]. Nevertheless, the overall utilization of these tests remains suboptimal in most countries, especially due to their invasiveness, cost, psychosocial barriers, or difficult accessibility [27]. For these reasons, there is a high demand for the development of blood‐based tests that could overcome some of these barriers. Today, several screening tests in various stages of development focus on the analysis of cell‐free DNA or methylated circulating tumor DNA, including multiomics tests using the combinations with proteins [28]. In recent years, many studies have addressed the diagnostic potential of noncoding RNAs, both in blood serum or plasma as well as in small EVs, as summarized in Shi et al. [29].

As small EVs may be released directly from tumor cells, thus reflecting the physiological and pathological status of tumors, we decided to perform a detailed analysis of the RNA profile of these vesicles in large cohorts of CRC patients and HCs to identify promising cancer‐associated molecules that could serve as biomarkers for early diagnosis. Importantly, the suitability of Clariom D Pico Assays for transcriptome profiling from a low volume of serum was confirmed in our previous study [22]. Overall, significantly different levels of 13 lncRNAs and four mRNAs were observed in the screening phase of the study and confirmed in an extensive and independent two‐phase validation. Importantly, the levels of the analyzed genes were greater in patients with metastatic disease than in patients with localized disease, suggesting that these molecules could be involved in the migration and formation of distant metastases. Importantly, the levels of several genes were also significantly different between the samples of HCs and PLs. Interestingly, they were often lower in patients with lesions than in HCs. This observation could be related to the fact that individual genes may be involved in the process of carcinogenesis differently in the early and later stages of the disease. Moreover, not all the molecules studied need to be associated directly with tumor tissue but may be released into small EVs by other cells in the body.

While most of the mRNAs studied in this work have been previously associated with cancer, almost none of the identified lncRNAs have been described thus far. Early growth response factor 1 (EGR1) is a zinc‐finger transcription factor induced by various stimuli including hypoxia, hyperglycemia, radiation, or chemotherapy [30]. It may be induced by TGF‐β and elevated levels are associated with enhanced tumor proliferation, angiogenesis, and invasiveness of CRC cells [31, 32]. In 2007, EGR1 was reported to be upregulated in the mucosa of CRC patients compared with HCs and associated with early onset CRC [33]. Furthermore, it plays an important role in the regulation of SIRT1‐dependent migration and invasion under hypoxia [30]. Importantly, the angiogenic activity of CRC cell‐derived EVs was associated with the activation of EGR1 in endothelial cells [34]. The CXCL12/CXCR4 axis plays a crucial role in tumor‐stromal cell communication and creates a favorable microenvironment for tumor growth and recruitment of CXCR4+ tumor cells to CXCL12‐rich stromal niches to initiate metastasis [35]. In breast cancer, the overexpression of CXCR4 in small EVs was associated with tumor recurrence and distant organ metastases [36]. Together with our results, it seems that EGR1 and CXCR4 might be actively secreted into small EVs by CRC tumor cells to subsequently participate in intercellular communication and provide an optimal microenvironment for the establishment of distant metastases. In contrast, the downregulation of regulator of G‐protein signaling 2 (RGS2) was observed in CRC tissues with recurrence and in metastasis‐derived cell lines, and its low levels correlated with poor patient survival [37]. Thus, targeted secretion of RGS2 into small EVs could serve as a defense mechanism of tumor cells against chemotherapy. Concerning the lncRNAs, SOS1‐IT1 has been previously described to be associated with worse prognosis of endometrial cancer patients through the regulation of autophagy [38] and hypoxia. Additionally, HIF1‐α was found to directly bind the SOS1‐IT1 promoter region and thus affect its expression [39]. Most of the dysregulated lncRNAs identified in this study are transcribed from the opposite strand of genes with protein‐coding functions. Growing evidence has demonstrated that antisense lncRNAs play crucial roles in tumor initiation and progression as well as chemotherapy resistance. Importantly, because of their sequence complementarity, these lncRNAs may regulate their corresponding sense genes [40]. In hepatocellular carcinoma, LASP1‐AS was described to function as an oncogene that promotes the proliferation and migration of tumor cells by increasing the expression of its sense‐cognate gene LASP1 [41]. In CRC, LASP1 is reported to enhance tumor progression and metastatic potential through the activation of the mitogen‐activated protein kinase signaling pathway [42], Hippo signaling pathway [43], or through interaction with the CCT8 [44]. Proteome profiling of small EVs derived from metastatic SW620 cells revealed the selective enrichment of lipid‐raft associated component flotillin‐2 (FLOT2), which regulates a number of biological processes, including cell adhesion, signal transduction, or actin cytoskeleton dynamics [45]. Increased levels of this protein are observed in CRC patients with metastatic disease and worse prognosis [46]. Mengwasser et al. [47] identified PHB1 as an antigen released from colorectal tumors *in vivo*, and blood serum from CRC patients contained significantly higher levels of PHB1 than did that from HCs. PAX5 is an important transcription factor that has been shown to activate the transcription of several important oncogenes involved in the pathogenesis of CRC, including the lncRNAs UASR1 [48] or SNHG25, which play critical roles in enhancing the metastatic capacity of CRC cells through the promotion of MMP2 expression [49]. Similarly, SMARCA4 (BRG1) is a well‐known transcription factor and a central component of the SWI/SNF chromatin‐remodeling complex. This protein is highly expressed in CRC tissue, and its high levels correlate with worse response to chemoradiation [50] and poor patient survival [51]. In addition, SMARCA4 may regulate the expression of VEGF‐A by interacting with HIF‐1α and thus promote angiogenesis [52]. Cysteine and glycine‐rich protein 1 (CSRP1) expression was associated with a mesenchymal, stromal‐rich tumor profile (CMS4) [53], increased proliferation, migration, and poor prognosis of CRC patients [54]. Recently, characterization of CRC cell lines through proteomic profiling of their EVs revealed that several key molecules involved in epithelial carcinogenesis, including PDPK1, are enriched in small EVs [55]. Although all of the abovementioned proteins have been previously described as important oncogenes involved in CRC initiation or progression, their association with corresponding antisense lncRNAs has not yet been described. Therefore, it is not possible to determine exactly the function of the described lncRNAs in the pathogenesis of the disease, whether they are able to regulate the expression of their sense protein‐coding genes, and how they contribute to the crosstalk between tumor and stromal cells in the microenvironment.

To further improve the diagnostic performance of identified molecules, we assessed a diagnostic panel consisting of seven lncRNAs and one mRNA that enabled us to differentiate patients from HCs with a sensitivity and specificity higher than 78% and the AUC > 0.85. Importantly, the ROC analysis confirmed its suitability also for the early diagnosis of CRC, and the specificity was significantly greater than that of FOBT. Similarly, the combined panel of three lncRNAs and three mRNAs distinguished HCs from patients with PLs with a sensitivity > 95% and a specificity of 86% in both phases of the study with an AUC > 0.97. To further test the association between CRC and dysregulated lncRNAs, we analyzed the levels of molecules of interest in samples from CRC patients before and 3 months after surgical resection of the tumor and in samples of tumor tissue and corresponding adjacent mucosa. We detected significantly decreased levels of seven molecules in postoperative samples. In addition, the expression of six genes was increased in CRC tumor tissue compared with adjacent mucosa. These data indicate the potential involvement of these molecules in CRC pathogenesis and their origin in tumor tissue.

We are also aware of several limitations of this study. First, there is currently no uniform methodology for the isolation and characterization of small EVs for the purpose of identifying novel diagnostic biomarkers. Thus, we used our previously optimized procedure [22]. Unfortunately, 150 μL of serum was insufficient to reliably detect the markers contained in small EVs via RT‐qPCR. For this reason, we were forced to pool the samples and subsequently perform DNA preamplification, which may have introduced some bias into the results. Second, protein marker analysis by western blot detected ApoB contamination in HCs. This result is probably because patients fast before surgery, whereas individuals undergoing preventive examinations may not meet this condition. Thus, the removal of lipoproteins from a blood sample prior to the isolation of small EVs might be included as a first step in the experimental procedure [56]. Third, no lncRNA that could serve as a suitable endogenous control for RT‐qPCR data normalization in CRC currently exists. For this reason, the combination of the GAPDH and ACTB genes, which have been used in previous studies [19, 57] and were confirmed to be suitable molecules via GeNorm/NormFinder algorithms, was used in this study. However, the identification of stably expressed lncRNAs associated with small EVs in CRC patients would certainly be preferable for future analyses. Finally, although we included a preamplification step, some lncRNAs still had high Ct values in several samples. Given the fact that a higher percentage of negative samples was observed in HCs, it is possible that the levels in these individuals were very low, which may be related to the absence of tumor cells that secrete these tumor‐associated lncRNAs into the circulation. However, the exact involvement of most of the studied lncRNAs in the pathogenesis of CRC has not yet been described, so it is not possible to determine whether they are secreted into small EVs as oncogenes involved in intercellular communication or whether they function as tumor suppressors that cancer cells want to eliminate.

## 5 Conclusions and future perspectives

This study highlights the diagnostic potential of lncRNAs and mRNAs isolated from serum‐derived small EVs. Using Clariom D Pico assays and subsequent RT‐qPCR validation, we have developed diagnostic panels distinguishing HCs from CRC patients and patients with PLs with high sensitivity and specificity. Several molecules also showed altered expression after tumor resection, indicating their tumor origin and relevance to CRC pathogenesis. Although many of the identified lncRNAs have not been previously described and their functional roles, especially in the context of EV‐mediated tumor‐stroma communication, remain largely unknown, some known oncogenes and tumor suppressors were also found enriched in small EVs of CRC patients, suggesting these vesicles may actively participate in metastasis formation and treatment resistance. Despite promising results, well‐designed prospective studies using higher input serum volume will be highly required to achieve more accurate detection in individual samples with the possibility of omitting the preamplification step, thus reducing bias. This approach would also allow analysis of potential associations between the levels of biomarkers and patient prognosis. In addition, identification of stably expressed lncRNAs in EVs is highly desirable to achieve the most accurate normalization of RT‐qPCR data. Finally, *in vitro* and *in vivo* studies to clarify the roles of individual lncRNAs in CRC progression, metastasis, and EV‐mediated signaling would also be beneficial. By addressing these areas, future research can contribute to the development of robust, noninvasive diagnostic tools for early CRC detection and monitoring.

## Conflict of interest

The authors declare no conflict of interest.

## Author contributions

PV‐F was involved in conceptualization, methodology, validation, writing – original draft, visualization; MP was involved in methodology, data acquisition—Small EVs and RNA isolation, RT‐qPCR validation; LP was involved in methodology, microarray expression profiling, small EVs and RNA isolation, RT‐qPCR validation; RJ was involved in microarray data analysis, formal analysis, resources, data curation; TM was involved in data acquisition, RT‐qPCR validation; LR was involved in biostatistical analysis—ROC analysis, DXscore, diagnostic panels; MS was involved in resources—blood serum collection, funding acquisition; RB, TS, ZF, JO, ES, EPM, DAT, MR were involved in processing of samples—small EVs and RNA isolation; MV was involved in resources—blood serum sample organization and retrieval; JK was involved in methodology, investigation—characterization of small EVs by DLS; TCI was involved in methodology—microarrays; MB was involved in methodology—WB analysis; LB was involved in resources, writing – review and editing; TS was involved in resources, data curation; JH was involved in data curation, project administration at MMCI; MS, VP, TG were involved in resources, data curation at Faculty Hospital Brno; ZK was involved in resources, project administration at Faculty Hospital Brno; OS was involved in supervision, writing – review and editing, funding acquisition. All the authors read and approved the final manuscript.

## Peer review

The peer review history for this article is available at https://www.webofscience.com/api/gateway/wos/peer‐review/10.1002/1878‐0261.70086.

## Ethics statement

Written informed consent was obtained from all participants, and the study was approved by the local Ethical Board at MMCI (2019/1842/MOU) as well as by the Research Ethics Committee at Masaryk University, Brno (EKV2019‐053) and conforms to recognized standards of the Helsinki Declaration.

## Supporting information


**Fig. S1.** Characterization of small extracellular vesicles via western blot analysis—raw western blot images.
**Fig. S2.** Hierarchical clustering—screening phase of the study.
**Fig. S3.** Significantly dysregulated long noncoding RNAs during the training phase of the study.
**Fig. S4.** Dysregulation of mRNAs during the training and validation phase of the study.
**Fig. S5.** Expression of long noncoding RNAs in samples of healthy controls, colorectal cancer patients and patients with precancerous lesions during the training phase of the study.
**Fig. S6.** Training phase of the study—correlation with clinicopathological characteristics.
**Fig. S7.** Performance of established long noncoding RNA‐based diagnostic panels.
**Fig. S8.** Significantly dysregulated long noncoding RNAs during the validation phase of the study.

**Table S1.** Clinicopathological characteristics of studied subjects.

**Table S2.** Clinicopathological characteristics of patients with gastric cancer.

**Table S3.** Clinicopathological characteristics of patients with colorectal cancer – tissue samples.

**Table S4.** List of primary antibodies used for western blot analysis.

**Table S5.** Sequences of IDT primers used in the training phase of the study.

**Table S6.** A list of RNA transcripts with significantly different levels between colorectal cancer patients and healthy controls as identified by microarray expression profiling (adjusted *P* < 0.05).

**Table S7.** Individual diagnostic performance of each candidate biomarker in the training and validation phase of the study—colorectal cancer patients vs. healthy controls.

**Table S8.** Individual diagnostic performance of each candidate biomarker in the training and validation phase of the study—healthy controls/colorectal cancer patients vs. precancerous lesions.

**Table S9.** Diagnostic performance of the established panels—colorectal cancer patients vs. healthy controls.

**Table S10.** Diagnostic performance of established panels—patients with precancerous lesions vs. healthy controls.

**Table S11.** Different levels of analyzed molecules in the validation phase of the study.

**Table S12.** Expression of potentially diagnostic long noncoding RNAs (lncRNAs) and mRNAs in paired samples (*n* = 50) of tumor tissue (TT) and adjacent mucosa (AM) from colorectal cancer patients.

## Data Availability

The microarray datasets generated and analyzed during the exploration phase of the study are available in the Gene Expression Omnibus database, GEO accession number GSE277272. (https://www.ncbi.nlm.nih.gov/geo/query/acc.cgi?acc=GSE277272). The other data that support the findings of our study are available from the corresponding author upon reasonable request.
