# Supplementary material for: Comprehensive profiling of lncRNAs and mRNAs enriched in small extracellular vesicles for early noninvasive detection of colorectal cancer: diagnostic panel assembly and extensive validation
Source: Mol Oncol. 2025 Jul 10;19(11):3445–62. doi: 10.1002/1878-0261.70086 (PMC12591314; doi:10.1002/1878-0261.70086)
Supplement: Supplementary file 1 — Fig. S1. Characterization of small extracellular vesicles via western blot analysis—raw western blot images. Fig. S2. Hierarchical clustering—screening phase of the study. Fig. S3. Significantly dysregulated long noncoding RNAs during the training phase of the study. Fig. S4. Dysregulation of mRNAs during the training and validation phase of the study. Fig. S5. Expression of long noncoding RNAs in samples of healthy controls, colorectal cancer patients and patients with precancerous lesions during the training phase of the study. Fig. S6. Training phase of the study—correlation with clinicopathological characteristics. Fig. S7. Performance of established long noncoding RNA‐based diagnostic panels. Fig. S8. Significantly dysregulated long noncoding RNAs during the validation phase of the study. [file MOL2-19-3445-s011.zip › MOLONC-25-0172_Supplementary_Figure_Legends.docx]

**Supplementary Figure S1: Characterization of small extracellular vesicles via western blot analysis – raw western blot images** (individual proteins were detected on different gels or different parts of a gel). CRC – representative samples of colorectal cancer (n = 3), HC - representative samples of healthy controls (n = 3), M – marker, HCT116 – colorectal cancer cell line

**Supplementary Figure S2:** **Hierarchical clustering – screening phase of the study.** Hierarchical clustering of 100 colorectal cancer (CRC) samples and 50 healthy control samples on the basis of the significantly different expression (adjusted *P* < 0.05) of mRNAs and/or long noncoding RNAs isolated from serum small extracellular vesicles during the screening phase of the study.

**Supplementary Figure S3:** **Significantly dysregulated long noncoding RNAs during the training phase of the study.** Expression of long noncoding RNAs isolated from serum small extracellular vesicles and significantly dysregulated between 66 pools of healthy controls (HC, green) and 66 pools of colorectal cancer patients (CRC, red) during the training phase of the study (two-tailed nonparametric Mann-Whitney U-test, *P* < 0.05). A box-and-whisker plot displays the minimum, first quartile, median, third quartile, and maximum values.

**Supplementary Figure S4:** **Dysregulation of mRNAs during the training and validation phase of the study.** Significant mRNAs isolated from serum small extracellular vesicles and dysregulated between (A) 66 pools of healthy controls (HC, green) and 66 pools of colorectal cancer patients (CRC, red) during the training phase of the study (two-tailed nonparametric Mann-Whitney U-test, *P* < 0.05). (B) 66 pools of HC (green) and 105 pools of CRC patients (red) during the validation phase of the study (two-tailed nonparametric Mann-Whitney U-test, *P* < 0.05). A box-and-whisker plot displays the minimum, first quartile, median, third quartile, and maximum values.

**Supplementary Figure S5:** **Expression of long noncoding RNAs in samples of healthy controls, colorectal cancer patients and patients with precancerous lesions during the training phase of the study.** Expression of 12 long noncoding RNAs isolated from serum small extracellular vesicles and significantly dysregulated between healthy controls (HC, n = 66, green) and patients with precancerous lesions (PL, n = 20, yellow) or patients with PL and colorectal cancer patients (CRC, n = 66, red) during the training phase of the study. Kruskal-Wallis test, **P* < 0.05; ***P* < 0.01; ****P* < 0.001. A box-and-whisker plot displays the minimum, first quartile, median, third quartile, and maximum values.

**Supplementary Figure S6:** **Training phase of the study – correlation with clinicopathological characteristics.** (A)-(C) Expression of mRNAs significantly dysregulated between the serum-derived small extracellular vesicles (EVs) of healthy controls (HC, n = 66, green) and patients with precancerous lesions (PL, n = 20, yellow) and/or patients with PL and colorectal cancer patients (CRC, n = 66, red). (D) The levels of CSRP1-AS increased progressively from stage I to stage IV (*P* = 0.0211, n = 66). (E)-(F) Significantly increased expression of EGR1 (*P* = 0.0330) and CXCR4 (*P* = 0.0385) in the small EVs of patients with metastatic disease (stage III+IV, n = 33, dark red) compared with patients with localized disease (stage I+II, n = 33, light red). (A)-(D) Kruskal-Wallis test, (E)-(F) two-tailed nonparametric Mann-Whitney U-test, **P* < 0.05; ****P* < 0.001. A box-and-whisker plot displays the minimum, first quartile, median, third quartile, and maximum values.

**Supplementary Figure S7:** **Performance of established long noncoding RNA-based diagnostic panels.** (A) ROC analysis using the expression of 7-lncRNA-based panel – the training cohort (AUC = 0.8104, n_CRC_ = 66, n_HC_ = 66). (B) ROC analysis using the expression of 3-lncRNA-based panel – the training cohort (AUC = 0.9220, n_PL_ = 20, n_HC_ = 66). (C) ROC analysis using the expression of 7-lncRNA-based panel – the validation cohort (AUC = 0.7879, n_CRC_ = 105, n_HC_ = 66). (D) ROC analysis using the expression of 3-lncRNA-based panel – the validation cohort (AUC = 0.9429, n_PL_ = 20, n_HC_ = 66). (E) ROC analysis using the expression of 7-lncRNA-based panel – the training cohort, stages I+II (AUC = 0.8085, n_CRC_ = 33, n_HC_ = 66). (F) ROC analysis using the expression of 7-lncRNA-based panel – the validation cohort, stages I+II (AUC = 0.8004, n_CRC_ = 59, n_HC_ = 66). CRC – colorectal cancer, HC – healthy control, PL – precancerous lesion, ROC – receiver operating characteristics, AUC – area under the curve. The red line corresponds to ROC curve.

**Supplementary Figure S8:** **Significantly dysregulated long noncoding RNAs during the validation phase of the study.** Long noncoding RNAs isolated from serum small extracellular vesicles and significantly dysregulated between 66 pools of healthy controls (HC, green) and 105 pools of colorectal cancer patients (CRC, red) during the validation phase of the study (two-tailed nonparametric Mann-Whitney U-test, *P* < 0.05). A box-and-whisker plot displays the minimum, first quartile, median, third quartile, and maximum values.
