# Supplementary material for: Comprehensive profiling of lncRNAs and mRNAs enriched in small extracellular vesicles for early noninvasive detection of colorectal cancer: diagnostic panel assembly and extensive validation
Source: Mol Oncol. 2025 Jul 10;19(11):3445–62. doi: 10.1002/1878-0261.70086 (PMC12591314; doi:10.1002/1878-0261.70086)
Supplement: Supplementary file 1 — Fig. S1. Characterization of small extracellular vesicles via western blot analysis—raw western blot images. Fig. S2. Hierarchical clustering—screening phase of the study. Fig. S3. Significantly dysregulated long noncoding RNAs during the training phase of the study. Fig. S4. Dysregulation of mRNAs during the training and validation phase of the study. Fig. S5. Expression of long noncoding RNAs in samples of healthy controls, colorectal cancer patients and patients with precancerous lesions during the training phase of the study. Fig. S6. Training phase of the study—correlation with clinicopathological characteristics. Fig. S7. Performance of established long noncoding RNA‐based diagnostic panels. Fig. S8. Significantly dysregulated long noncoding RNAs during the validation phase of the study. [file MOL2-19-3445-s011.zip › Supplementary Figure S3.pdf]

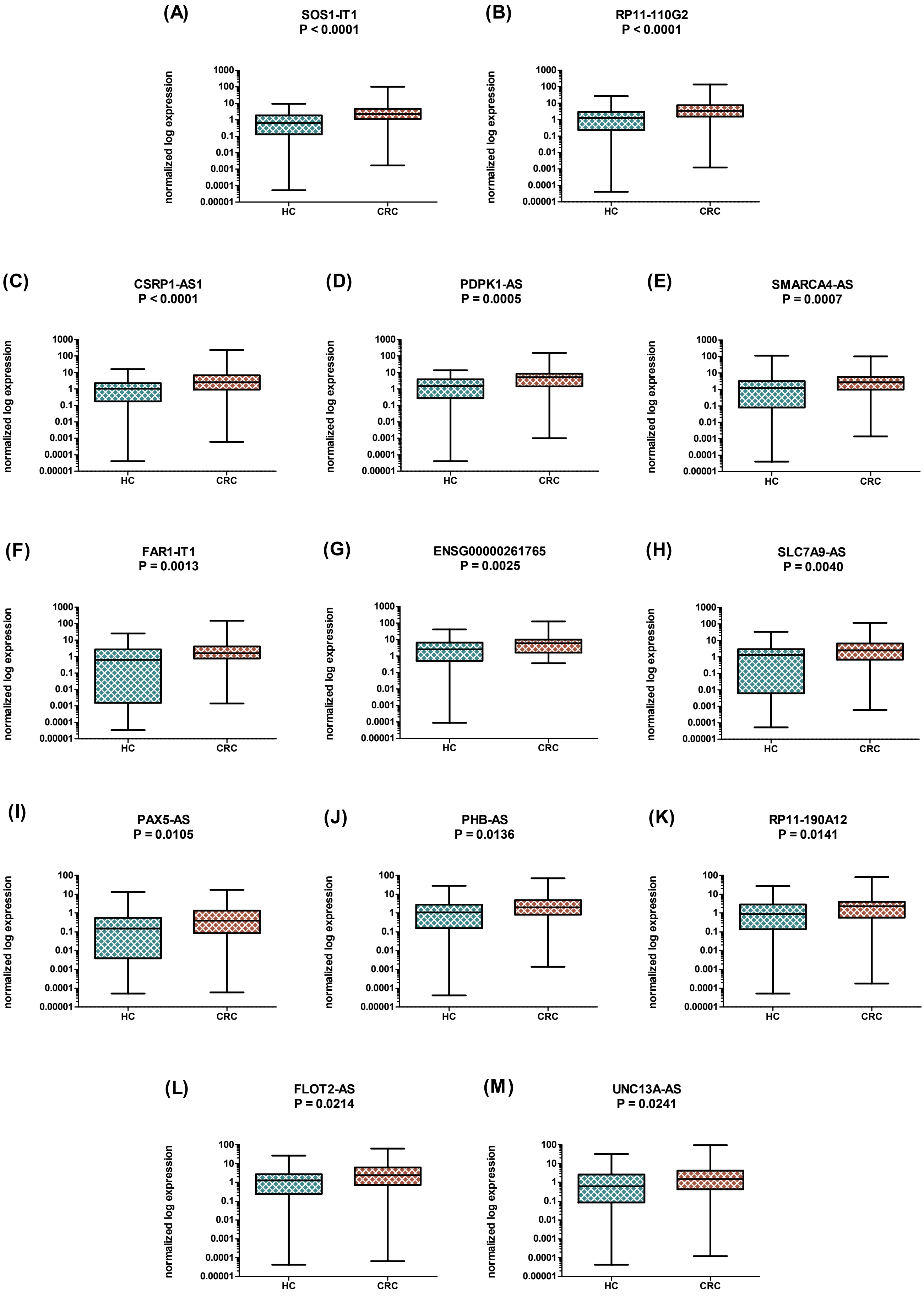

(A)
SOS1-IT1
P < 0.0001
(B)
RP11-110G2
P < 0.0001
(C)
CSRP1-AS1
P < 0.0001
(D)
PDPK1-AS
P = 0.0005
(E)
SMARCA4-AS
P = 0.0007
(F)
FAR1-IT1
P = 0.0013
(G)
ENSG00000261765
P = 0.0025
(H)
SLC7A9-AS
P = 0.0040
(I)
PAX5-AS
P = 0.0105
(J)
PHB-AS
P = 0.0136
(K)
RP11-190A12
P = 0.0141
(L)
FLOT2-AS
P = 0.0214
(M)
UNC13A-AS
P = 0.0241
normalized log expression
1000
100
10
1
0.1
0.01
0.001
0.0001
0.00001
HC
CRC
