# Supplementary material for: Comprehensive profiling of lncRNAs and mRNAs enriched in small extracellular vesicles for early noninvasive detection of colorectal cancer: diagnostic panel assembly and extensive validation
Source: Mol Oncol. 2025 Jul 10;19(11):3445–62. doi: 10.1002/1878-0261.70086 (PMC12591314; doi:10.1002/1878-0261.70086)
Supplement: Supplementary file 2 — Table S1. Clinicopathological characteristics of studied subjects. [file MOL2-19-3445-s008.docx]

**Supplementary Table S1:** Clinicopathological characteristics of studied subjects.

| **CRC cases** | **Screening phase** | **Training phase** | **Validation phase** |
| --- | --- | --- | --- |
| **Number** | 100 | 198 | 315 |
| **Age (mean ± s.d.)*, years** | 67 ± 12 | 65 ± 12 | 65 ± 12 |
| **Sex, number (%)** | | | |
| Male | 62 (62) | 123 (62) | 174 (55) |
| Female | 38 (38) | 75 (38) | 141 (45) |
| **Diagnosis, number (%)** |  |  |  |
| C18 | 50 (50) | 102 (52) | 234 (74) |
| C19 | 20 (20) | 39 (19) | 21 (7) |
| C20 | 30 (30) | 57 (29) | 60 (19) |
| **TNM stage, number (%)** | | | |
| Stage I | 20 (20) | 36 (18) | 57 (18) |
| Stage II | 30 (30) | 63 (32) | 123 (39) |
| Stage III | 30 (30) | 60 (30) | 69 (22) |
| Stage IV | 20 (20) | 39 (20) | 66 (21) |
| Unknown | - | - | - |
| **Grade, number (%)** | | | |
| Grade 1 | 8 (8) | 46 (23) | 60 (19) |
| Grade 2 | 68 (68) | 109 (55) | 176 (56) |
| Grade 3 | 17 (17) | 34 (17) | 68 (22) |
| Grade 4 | 2 (2) | 1 (1) | 1 (0) |
| Unknown | 5 (5) | 8 (4) | 10 (3) |
| **Location, number (%)** | | | |
| Distal | 72 (72) | 142 (72) | 186 (59) |
| Proximal | 28 (28) | 55 (28) | 124 (39) |
| Unknown | 0 (0) | 1 (0) | 5 (2) |
| **Tumor size, number (%)** | | | |
| < 50 mm | 41 (41) | 110 (56) | 159 (51) |
| ≥ 50 mm | 38 (38) | 80 (40) | 143 (45) |
| Unknown | 21 (21) | 8 (4) | 13 (4) |
| **Healthy controls** | **Screening phase** | **Training phase** | **Validation phase** |
| **Number** | 50 | 198 | 198 |
| **Age (mean ± s.d.), years** | 66 ± 5 | 59 ± 7 | 63 ± 7 |
| **Sex, number (%)** |  |  |  |
| Male | 29 (58) | 117 (59) | 117 (59) |
| Female | 21 (42) | 81 (41) | 81 (41) |
| **Precancerous lesions** | **Screening phase** | **Training phase** | **Validation phase** |
| **Number** | - | 60 | 60 |
| **Age (mean ± s.d.), years** | - | 63 ± 11 | 63 ± 12 |
| **Sex, number (%)** |  |  |  |
| Male | - | 36 (60) | 31 (52) |
| Female | - | 24 (40) | 29 (48) |
| **Histology, number (%)** |  |  |  |
| Tubular | - | 30 (50) | 30 (50) |
| Tubulovillous | - | 3 (5) | 6 (10) |
| Hyperplastic | - | 15 (25) | 15 (25) |
| Serrated | - | 6 (10) | 3 (5) |
| More types | - | 6 (10) | 6 (10) |

*s.d. – standard deviation
