# Supplementary material for: Comprehensive profiling of lncRNAs and mRNAs enriched in small extracellular vesicles for early noninvasive detection of colorectal cancer: diagnostic panel assembly and extensive validation
Source: Mol Oncol. 2025 Jul 10;19(11):3445–62. doi: 10.1002/1878-0261.70086 (PMC12591314; doi:10.1002/1878-0261.70086)
Supplement: Supplementary file 3 — Table S2. Clinicopathological characteristics of patients with gastric cancer. [file MOL2-19-3445-s005.docx]

**Supplementary Table S2:** Clinicopathological characteristics of patients with gastric cancer.

| **Characteristics** | **blood serum** |
| --- | --- |
| **Number** | 54 |
| **Age (mean ± s.d.)*, years** | 65 ± 14 |
| **Sex, number (%)** |  |
| Male | 30 (56) |
| Female | 24 (44) |
| **TNM stage, number (%)** |  |
| Stage I | 9 (17) |
| Stage II | 15 (28) |
| Stage III | 24 (44) |
| Stage IV | 6 (11) |
| **Location, number (%)** |  |
| Fundus | 4 (7) |
| Cardia | 6 (11) |
| Body | 12 (22) |
| Lesser curvature | 10 (19) |
| Greature curvature | 4 (7) |
| Antrum | 16 (30) |
| Pylorus | 2 (4) |

*s.d. – standard deviation
