# Supplementary material for: Comprehensive profiling of lncRNAs and mRNAs enriched in small extracellular vesicles for early noninvasive detection of colorectal cancer: diagnostic panel assembly and extensive validation
Source: Mol Oncol. 2025 Jul 10;19(11):3445–62. doi: 10.1002/1878-0261.70086 (PMC12591314; doi:10.1002/1878-0261.70086)
Supplement: Supplementary file 4 — Table S3. Clinicopathological characteristics of patients with colorectal cancer – tissue samples. [file MOL2-19-3445-s003.docx]

**Supplementary Table S3:** Clinicopathological characteristics of patients with colorectal cancer – tissue samples.

| **Characteristics** | **Tumor tissue** |
| --- | --- |
| **Number** | 50 |
| **Age (mean ± s.d.)*, years** | 62 ± 12 |
| **Sex, number (%)** |  |
| Male | 34 (68) |
| Female | 16 (32) |
| **Diagnosis, number (%)** |  |
| C18 | 36 (72) |
| C19 | 14 (28) |
| **TNM stage, number (%)** |  |
| Stage I | 9 (18) |
| Stage II | 12 (24) |
| Stage III | 12 (24) |
| Stage IV | 17 (34) |
| **Grade, number (%)** |  |
| Grade 1 | 10 (20) |
| Grade 2 | 31 (62) |
| Grade 3 | 8 (16) |
| Unknown | 1 (2) |
| **Tumor size, number (%)** |  |
| < 50 mm | 24 (48) |
| ≥ 50 mm | 16 (32) |
| Unknown | 10 (20) |

*s.d. – standard deviation
