# Supplementary material for: Comprehensive profiling of lncRNAs and mRNAs enriched in small extracellular vesicles for early noninvasive detection of colorectal cancer: diagnostic panel assembly and extensive validation
Source: Mol Oncol. 2025 Jul 10;19(11):3445–62. doi: 10.1002/1878-0261.70086 (PMC12591314; doi:10.1002/1878-0261.70086)
Supplement: Supplementary file 5 — Table S4. List of primary antibodies used for western blot analysis. [file MOL2-19-3445-s013.docx]

**Supplementary Table S4:** A list of primary antibodies used for western blot analysis.

| **Target** | **Dilution** | **Origin** | **Catalog number** | **Producer** |
| --- | --- | --- | --- | --- |
| CD81 | 1:500 | mouse | sc-166029 | Santa Cruz Biotechnology, Dallas, Texas, USA |
| ApoB | 1:5000 | mouse | sc-13538 | Santa Cruz Biotechnology, Dallas, Texas, USA |
| TSG101 | 1:200 | mouse | 612696 | BD Biosciences, Franklin Lakes, NJ, USA |
| Syntenin | 1:1000 | rabbit | ab133267 | Abcam, Cambridge, UK |
| ACTB | 1:5000 | rabbit | 4970 | Cell Signaling, Boston, MA, USA |

HSP70 – heat shock protein 70, ApoB – apolipoprotein B, TSG101 – tumor susceptibility gene 101
