# Supplementary material for: Comprehensive profiling of lncRNAs and mRNAs enriched in small extracellular vesicles for early noninvasive detection of colorectal cancer: diagnostic panel assembly and extensive validation
Source: Mol Oncol. 2025 Jul 10;19(11):3445–62. doi: 10.1002/1878-0261.70086 (PMC12591314; doi:10.1002/1878-0261.70086)
Supplement: Supplementary file 6 — Table S5. Sequences of IDT primers used in the training phase of the study. [file MOL2-19-3445-s001.docx]

**Supplementary Table S5:** Sequences of IDT primers used in the training phase of the study.

| **Ensembl ID** | **Gene name** | **Forward primer (5’-3’)** | **Reverse primer (5’-3’)** |
| --- | --- | --- | --- |
| ENSG00000259215 | LINC02896 | GGACCTACTAGACCTGGAGATT | GGAATGAAAGAGGTAAGTGAACATT |
| ENSG00000229692 | SOS1-IT1 | TCGATGTGCTAATCAAGTTCCC | GTTAGTGCTTCTCAACCTCCTC |
| ENSG00000263466 | LASP1-AS | CCATAGCATGGACCCATCAAA | CTGAGAATGTGTGGAAGTGAGG |
| ENSG00000259563 | CTDSPL2-AS | TGGAAACCCTCAAGTGTGTAG | TCCCTCTTGGAGCTTTCATTTA |
| ENSG00000260558 | ENSG00000260558 | TAAGCTAAGCTACGGTTGGAAG | CTCCTCATGGCACAGGTAAA |
| ENSG00000258885 | PAX5-AS | ACACACCTGGCTGAGAGAT | CCCTCTTAGGTGGTTTCTTCAC |
| ENSG00000267555 | SLC7A9-AS | ACCCAGAGGTGTACAGTGA | ACCTGTCCTGACTCCAAGA |
| ENSG00000257298 | LIMA1-SI | CTGGCTAGTTAGAAGCTCAAGG | CCACATCGGCTCTCTCTTTAAT |
| ENSG00000261140 | PDPK1-AS | GTGCCCAAGCTATTTCACTATTT | GTGACGTGGGTAGAGTTTCTT |
| ENSG00000266936 | SMARCA4-AS | GCTGTTCTTTGGTTGGAGTTG | CTTGGGACAGTCACGAAACA |
| ENSG00000264304 | FLOT2-AS | GCCAGTTAGGTGCCTCTTC | GGAAAGCAAGTTGGGTTAGAAAG |
| ENSG00000254791 | FAR1-IT1 | GTAAGAGGCACCTACTGAAACC | GCGTTTGCTGCTTGTATACTTT |
| ENSG00000224536 | CSRP1-AS1 | CCCACAAAGATGGCAAAGATG | GTTGGTACTTCAGTCCTCTCTTATC |
| ENSG00000261765 | ENSG00000261765 | GAGCCTGCATTTCATTACCTTT | CACGGAAGCAGTGAATGAATATC |
| ENSG00000287040 | RP11-190A12 | CCAGCCGACTCTTTCTTTCA | GAGAGGACTGATGCCAAGTTT |
| ENSG00000258693 | ENSG00000258693 | ATGACCTGAGCTAAACTGCTATT | AGATTATCTCTGCCAGTTTGTGA |
| ENSG00000231336 | RP11-110G2 | TGTCCAGAGTCAAAGATGCTC | CCTGAAGTGGTAGCAGTCAC |
| ENSG00000269752 | UNC13A-AS | CTGGTCTCAAACTCCTGACTTC | TCAAGCAGACAAGGGAACTTT |
| ENSG00000267138 | APBA3-SI | CCACCATACCTGGCTAAGAAAT | GGCAACAAGGTGAGACTGT |
| ENSG00000262039 | PHB-AS | ACGTCAACTCAATACCCATCC | GTCCACCTCTTCATTGTCCTAC |
| ENSG00000213402 | PTPRCAP | GACACAGACTATGACCACGTC | TCACTGTCTCTGGCTTCCT |
| ENSG00000253506 | NACA2 | GGTCTGGAACAGCATCTGATAG | CCGACTGGTTCTTCATCAATTTC |
| ENSG00000120738 | EGR1 | ACGCCGAACACTGACATTT | CTGCTGTGGAAACAGGTAGTC |
| ENSG00000117091 | CD48 | GATACCTGGCGAGTCTGTAAAC | CTTGGCAAGTATAACACCTGGA |
| ENSG00000136156 | ITM2B | TGTCATCTTAAATGAGCCCTCTG | GGGACAGGCACACTGATAAA |
| ENSG00000197061 | H4C3 | GGAAGGGTGGTGCTAAGCG | CCGAGCCAAACGGCGAATA |
| ENSG00000116741 | RGS2 | GGGTATACAGCTTGATGGAGAA | ATGTAGCATGAGGCTCTGTG |
| ENSG00000121966 | CXCR4 | CTCCTTCATCCTCCTGGAAATC | TGGCTCCAAGGAAAGCATAG |
