# Supplementary material for: Comprehensive profiling of lncRNAs and mRNAs enriched in small extracellular vesicles for early noninvasive detection of colorectal cancer: diagnostic panel assembly and extensive validation
Source: Mol Oncol. 2025 Jul 10;19(11):3445–62. doi: 10.1002/1878-0261.70086 (PMC12591314; doi:10.1002/1878-0261.70086)
Supplement: Supplementary file 8 — Table S7. Individual diagnostic performance of each candidate biomarker in the training and validation phase of the study—colorectal cancer patients vs. healthy controls. [file MOL2-19-3445-s007.docx]

**Supplementary Table S7:** The individual diagnostic performance of each candidate biomarker in the training and validation phase of the study – colorectal cancer patients vs. healthy controls.

|  | **Training phase** | | | **Validation phase** | | | |
| --- | --- | --- | --- | --- | --- | --- | --- |
| **Gene name** | **AUC** | ***P*-value** | **95% CI** | | **AUC** | ***P*-value** | **95% CI** |
| **SOS1-IT1** | 0.7225 | < 0.0001 | 0.6351-0.8100 | | 0.7400 | < 0.0001 | 0.6640-0.8160 |
| **RP11-110G2** | 0.7091 | < 0.0001 | 0.6206-0.7975 | | 0.7675 | < 0.0001 | 0.6949-0.8401 |
| **CSRP1-AS1** | 0.6997 | < 0.0001 | 0.6111-0.7883 | | 0.7270 | < 0.0001 | 0.6498-0.8043 |
| **PDPK1-AS** | 0.6768 | 0.00046 | 0.5848-0.7687 | | 0.7358 | < 0.0001 | 0.6599-0.8177 |
| **SMARCA4-AS** | 0.6716 | 0.00071 | 0.5784-0.7647 | | 0.7272 | < 0.0001 | 0.6497-0.8047 |
| **FAR1-IT1** | 0.6628 | 0.00126 | 0.5696-0.7559 | | 0.6660 | 0.00035 | 0.5837-0.7484 |
| **ENSG00000261765** | 0.6531 | 0.00250 | 0.5598-0.7465 | | 0.6927 | < 0.0001 | 0.6125-0.7728 |
| **SLC7A9-AS** | 0.6453 | 0.00398 | 0.5510-0.7397 | | 0.6807 | < 0.0001 | 0.5992-0.7621 |
| **PAX5-AS** | 0.6292 | 0.01043 | 0.5341-0.7243 | | 0.6772 | 0.00013 | 0.5955-0.7590 |
| **PHB-AS** | 0.6247 | 0.01350 | 0.5294-0.7199 | | 0.7491 | < 0.0001 | 0.6740-0.8243 |
| **RP11-190A12** | 0.6240 | 0.01402 | 0.5279-0.7200 | | 0.7419 | < 0.0001 | 0.6664-0.8175 |
| **FLOT2-AS** | 0.6162 | 0.02133 | 0.5200-0.7123 | | 0.7845 | < 0.0001 | 0.7141-0.8548 |
| **UNC13A-AS** | 0.6139 | 0.02403 | 0.5172-0.7105 | | 0.7155 | < 0.0001 | 0.6367-0.7944 |
| **EGR1** | 0.7772 | < 0.0001 | 0.6969-0.8574 | | 0.8051 | < 0.0001 | 0.7374-0.8729 |
| **RGS2** | 0.7325 | < 0.0001 | 0.6464-0.8186 | | 0.8380 | < 0.0001 | 0.7765-0.8995 |
| **CXCR4** | 0.7198 | < 0.0001 | 0.6282-0.8113 | | 0.7588 | < 0.0001 | 0.6851-0.8325 |
| **ITM2B** | 0.6974 | 0.00011 | 0.6066-0.7881 | | 0.7575 | < 0.0001 | 0.6814-0.8336 |
| **PTPRCAP** | 0.6373 | 0.00785 | 0.5408-0.7339 | | NA | NA | NA |

AUC – area under the curve, CI – confidence interval, NA – not analyzed
