# Supplementary material for: Comprehensive profiling of lncRNAs and mRNAs enriched in small extracellular vesicles for early noninvasive detection of colorectal cancer: diagnostic panel assembly and extensive validation
Source: Mol Oncol. 2025 Jul 10;19(11):3445–62. doi: 10.1002/1878-0261.70086 (PMC12591314; doi:10.1002/1878-0261.70086)
Supplement: Supplementary file 9 — Table S8. Individual diagnostic performance of each candidate biomarker in the training and validation phase of the study—healthy controls/colorectal cancer patients vs. precancerous lesions. [file MOL2-19-3445-s004.docx]

**Supplementary Table S8:** The individual diagnostic performance of each candidate biomarker in the training and validation phase of the study – healthy controls/colorectal cancer patients vs. precancerous lesions.

|  | **HEALTHY CONTROLS vs. PRECANCEROUS LESIONS** | | | | | |
| --- | --- | --- | --- | --- | --- | --- |
|  | **Training phase** | | | **Validation phase** | | |
| **Gene name** | **AUC** | ***P*-value** | **95% CI** | **AUC** | ***P*-value** | **95% CI** |
| **SOS1-IT1** | 0.7333 | 0.00165 | 0.6224-0.8443 | 0.6365 | 0.06709 | 0.4904-0.7826 |
| **LASP1-AS** | 0.5894 | 0.22780 | 0.4608-0.7180 | 0.8595 | < 0.0001 | 0.7564-0.9627 |
| **FLOT2-AS** | 0.5758 | 0.30670 | 0.4527-0.6988 | 0.7119 | 0.00449 | 0.5705-0.8533 |
| **PHB-AS** | 0.5424 | 0.56700 | 0.4126-0.6723 | 0.6849 | 0.01314 | 0.5516-0.8182 |
| **UNC13A-AS** | 0.5182 | 0.80620 | 0.3862-0.6502 | 0.6841 | 0.01353 | 0.5506-0.8176 |
| **RGS2** | 0.7069 | 0.00534 | 0.5878-0.8261 | 0.8095 | < 0.0001 | 0.6786-0.9405 |
| **EGR1** | 0.6653 | 0.02690 | 0.5406-0.7900 | 0.9183 | < 0.0001 | 0.8430-0.9935 |
| **PTPRCAP** | 0.6242 | 0.09640 | 0.4937-0.7547 | 0.8611 | < 0.0001 | 0.7611-0.9611 |
|  | **COLORECTAL CANCER vs. PRECANCEROUS LESIONS** | | | | | |
|  | **Training phase** | | | **Validation phase** | | |
| **Gene name** | **AUC** | ***P*-value** | **95% CI** | **AUC** | ***P*-value** | **95% CI** |
| **ENSG00000261765** | 0.7662 | 0.00034 | 0.6478-0.8845 | NA | NA | NA |
| **SMARCA4-AS** | 0.7585 | 0.00050 | 0.6471-0.8698 | NA | NA | NA |
| **RP11-110G2** | 0.7308 | 0.00189 | 0.6159-0.8456 | NA | NA | NA |
| **PDPK1-AS** | 0.7197 | 0.00305 | 0.6080-0.8314 | NA | NA | NA |
| **RP11-190A12** | 0.7136 | 0.00396 | 0.5937-0.8335 | NA | NA | NA |
| **FLOT2-AS** | 0.7129 | 0.00409 | 0.6032-0.8226 | 0.8716 | < 0.0001 | 0.7885-0.9546 |
| **CSRP1-AS1** | 0.7000 | 0.00699 | 0.5815-0.8185 | NA | NA | NA |
| **APBA3-SI** | 0.6985 | 0.00743 | 0.5822-0.8148 | NA | NA | NA |
| **PHB-AS** | 0.6917 | 0.00973 | 0.5664-0.8170 | 0.8520 | < 0.0001 | 0.7619-0.9420 |
| **LASP1-AS** | 0.6879 | 0.01127 | 0.5671-0.8086 | NA | NA | NA |
| **SLC7A9-AS** | 0.6871 | 0.01160 | 0.5654-0.8089 | NA | NA | NA |
| **UNC13A-AS** | 0.6394 | 0.06005 | 0.5163-0.7625 | 0.8361 | < 0.0001 | 0.7474-0.9248 |
| **SOS1-IT1** | 0.5219 | 0.76870 | 0.3821-0.6617 | 0.8000 | < 0.0001 | 0.7009-0.8991 |
| **EGR1** | 0.9254 | < 0.0001 | 0.8610-0.9898 | 0.9787 | < 0.0001 | 0.9519-1.006 |
| **RGS2** | 0.9231 | < 0.0001 | 0.8486-0.9976 | 0.9436 | < 0.0001 | 0.8922-0.9949 |
| **CXCR4** | 0.8405 | < 0.0001 | 0.7412-0.9397 | NA | NA | NA |
| **ITM2B** | 0.7133 | 0.00416 | 0.6074-0.8191 | 0.7817 | < 0.0001 | 0.6587-0.9046 |

AUC – area under the curve, CI – confidence interval, NA – not analyzed
