# Supplementary material for: Comprehensive profiling of lncRNAs and mRNAs enriched in small extracellular vesicles for early noninvasive detection of colorectal cancer: diagnostic panel assembly and extensive validation
Source: Mol Oncol. 2025 Jul 10;19(11):3445–62. doi: 10.1002/1878-0261.70086 (PMC12591314; doi:10.1002/1878-0261.70086)
Supplement: Supplementary file 10 — Table S9. Diagnostic performance of the established panels—colorectal cancer patients vs. healthy controls. [file MOL2-19-3445-s002.docx]

**Supplementary Table S9:** Diagnostic performance of the established panels – colorectal cancer patients vs. healthy controls.

| **PANEL A – lncRNAs-based** | | | | |
| --- | --- | --- | --- | --- |
|  | **Training cohort** | **Training cohort**  **stages I + II** | **Validation cohort** | **Validation cohort**  **stages I+II** |
| **AUC^†^** | 0.810 | 0.809 | 0.788 | 0.800 |
| **Sensitivity** | 0.758 | 0.758 | 0.735 | 0.746 |
| **Specificity** | 0.712 | 0.727 | 0.698 | 0.698 |
| **Accuracy** | 0.735 | 0.747 | 0.721 | 0.721 |
| **PPV^#^** | 0.746 | 0.600 | 0.798 | 0.698 |
| **NPV^##^** | 0.725 | 0.847 | 0.620 | 0.746 |
| **PANEL B – lncRNAs/mRNA-based** | | | | |
|  | **Training cohort** | **Training cohort**  **stages I + II** | **Validation cohort** | **Validation cohort**  **stages I+II** |
| **AUC^†^** | 0.854 | 0.862 | 0.852 | 0.864 |
| **Sensitivity** | 0.833 | 0.849 | 0.784 | 0.814 |
| **Specificity** | 0.788 | 0.788 | 0.778 | 0.778 |
| **Accuracy** | 0.811 | 0.808 | 0.782 | 0.795 |
| **PPV^#^** | 0.797 | 0.667 | 0.851 | 0.774 |
| **NPV^##^** | 0.825 | 0.847 | 0.690 | 0.817 |

**^†^**AUC = area under the curve, ^#^PPV- positive predictive value, ^##^NPV – negative predictive value
