# Supplementary material for: Comprehensive profiling of lncRNAs and mRNAs enriched in small extracellular vesicles for early noninvasive detection of colorectal cancer: diagnostic panel assembly and extensive validation
Source: Mol Oncol. 2025 Jul 10;19(11):3445–62. doi: 10.1002/1878-0261.70086 (PMC12591314; doi:10.1002/1878-0261.70086)
Supplement: Supplementary file 11 — Table S10. Diagnostic performance of established panels—patients with precancerous lesions vs. healthy controls. [file MOL2-19-3445-s009.docx]

**Supplementary Table S10:** Diagnostic performance of established panels – patients with precancerous lesions vs. healthy controls.

| **PANEL A – lncRNAs-based** | | | **PANEL B – lncRNAs/mRNAs-based** | | |
| --- | --- | --- | --- | --- | --- |
|  | **Training cohort** | **Validation cohort** | **Training cohort** | | **Validation cohort** |
| **AUC^†^** | 0.922 | 0.943 | 0.977 | 0.983 | |
| **Sensitivity** | 0.900 | 0.850 | 1.000 | 0.950 | |
| **Specificity** | 0.849 | 0.905 | 0.864 | 0.952 | |
| **Accuracy** | 0.860 | 0.892 | 0.895 | 0.952 | |
| **PPV^#^** | 0.643 | 0.739 | 0.714 | 0.864 | |
| **NPV^##^** | 0.966 | 0.950 | 1.000 | 0.984 | |

**^†^**AUC = area under the curve, ^#^PPV- positive predictive value, ^##^NPV – negative predictive value
