# Supplementary material for: Comprehensive profiling of lncRNAs and mRNAs enriched in small extracellular vesicles for early noninvasive detection of colorectal cancer: diagnostic panel assembly and extensive validation
Source: Mol Oncol. 2025 Jul 10;19(11):3445–62. doi: 10.1002/1878-0261.70086 (PMC12591314; doi:10.1002/1878-0261.70086)
Supplement: Supplementary file 12 — Table S11. Different levels of analyzed molecules in the validation phase of the study. [file MOL2-19-3445-s006.docx]

**Supplementary Table S11:** Different levels of analyzed molecules in the validation phase of the study.

| **A) PRECANCEROUS LESIONS vs HEALTHY CONTROLS** | | | | | |
| --- | --- | --- | --- | --- | --- |
| **Gene biotype** | **Gene name** | **Median FC (PL vs. HC)** | **Median PL levels (min-max)** | **Median HC levels (min-max)** | ***P*-value** |
| lncRNA | **LASP1-AS** | 0.23 | 1.12 (0.37-11.32) | 4.91 (0.61-58.04) | **< 0.0001** |
| lncRNA | **FLOT2-AS** | 0.50 | 3.16 (0.40-34.41) | 6.35 (0.90-67.82) | **0.0045** |
| lncRNA | **PHB-AS** | 0.55 | 4.17 (1.06-46.00) | 7.64 (0.91-89.56) | **0.0133** |
| lncRNA | **UNC13A-AS** | 0.57 | 2.63 (0.57-19.47) | 4.59 (0.71-52.35) | **0.0280** |
| lncRNA | SOS1-IT1 | 0.77 | 3.81 (1.03-23.75) | 4.94 (0.67-52.80) | 0.1239 |
| mRNA | **EGR1** | 0.24 | 4.14 (1.04-24.84) | 17.53 (3.58-165.00) | **< 0.0001** |
| mRNA | **PTPRCAP** | 0.39 | 5.64 (1.37-31.37) | 14.54 (2.71-89.37) | **< 0.0001** |
| mRNA | **RGS2** | 0.41 | 8.31 (2.17-41.32) | 20.23 (6.85-81.30) | **< 0.0001** |
| mRNA | ITM2B | 0.95 | 16.39 (3.24-125.10) | 17.34 (7.89-71.05) | 0.3460 |
| **B) PRECANCEROUS LESIONS vs COLORECTAL CANCER** | | | | | |
| **Gene biotype** | **Gene name** | **Median FC (PL vs. CRC)** | **Median PL levels (min-max)** | **Median CRC levels (min-max)** | ***P*-value** |
| lncRNA | **FLOT2-AS** | 0.17 | 3.16 (0.40-34.41) | 18.29 (1.25-258.00) | **< 0.0001** |
| lncRNA | **PHB-AS** | 0.22 | 4.17 (1.06-46.00) | 19.00 (1.49-259.40) | **< 0.0001** |
| lncRNA | **UNC13A-AS** | 0.28 | 2.63 (0.57-19.47) | 9.41 (0.46-126.20) | **< 0.0001** |
| lncRNA | **SOS1-IT1** | 0.28 | 3.81 (1.03-23.75) | 13.61 (0.50-171.20) | **< 0.0001** |
| mRNA | **EGR1** | 0.10 | 4.14 (1.04-24.84) | 43.22 (0.85-384.40) | **< 0.0001** |
| mRNA | **RGS2** | 0.17 | 8.31 (2.17-41.32) | 47.84 (2.78-317.70) | **< 0.0001** |
| mRNA | **ITM2B** | 0.52 | 16.39 (3.24-125.10) | 31.44 (1.57-237.80) | **0.0001** |

Different levels of long noncoding RNAs and mRNAs in small extracellular vesicles isolated from the blood serum of patients with precancerous lesions (PL) compared with those of A) healthy controls (HC), B) colorectal cancer patients (CRC). FC – fold change, *P*-values in bold are statistically significant.
