# Supplementary material for: Comprehensive profiling of lncRNAs and mRNAs enriched in small extracellular vesicles for early noninvasive detection of colorectal cancer: diagnostic panel assembly and extensive validation
Source: Mol Oncol. 2025 Jul 10;19(11):3445–62. doi: 10.1002/1878-0261.70086 (PMC12591314; doi:10.1002/1878-0261.70086)
Supplement: Supplementary file 13 — Table S12. Expression of potentially diagnostic long noncoding RNAs (lncRNAs) and mRNAs in paired samples (n = 50) of tumor tissue (TT) and adjacent mucosa (AM) from colorectal cancer patients. [file MOL2-19-3445-s012.docx]

**Supplementary Table S12:** Expression of potentially diagnostic long noncoding RNAs (lncRNAs) and mRNAs in paired samples (n = 50) of tumor tissue (TT) and adjacent mucosa (AM) from colorectal cancer patients.

| **Gene biotype** | **Gene name** | **Median FC**  **(TT vs. AM)** | **Median TT expression (min-max)** | **Median AM expression**  **(min-max)** | ***P*-value*** |
| --- | --- | --- | --- | --- | --- |
| lncRNA | **PDPK1-AS** | 2.07 | 5.30 (0.93-55.34) | 2.92 (0.33-19.69) | **< 0.0001** |
| lncRNA | **SOS1-IT1** | 1.86 | 1.44 (0.32-14.69) | 0.94 (0.10-8.06) | **0.0001** |
| lncRNA | **SMARCA4-AS** | 2.19 | 1.34 (0.24-24.21) | 0.62 (0.05-11.17) | **0.0004** |
| lncRNA | **FLOT2-AS** | 1.95 | 2.07 (0.29-28.52) | 1.04 (0.10-12.69) | **0.0016** |
| lncRNA | **CSRP1-AS1** | 1.33 | 1.52 (0.41-31.30) | 1.14 (0.11-15.86) | **0.0162** |
| lncRNA | FAR1-IT1 | 1.30 | 1.64 (0.31-33.87) | 1.42 (0.24-20.18) | 0.1303 |
| lncRNA | RP11-110G2 | 0.97 | 0.33 (0.07-27.26) | 0.33 (0.03-13.97) | 0.4327 |
| lncRNA | PAX5-AS | 0.43 | 0.10 (0.00-22.38) | 0.15 (0.01-10.84) | 0.5417 |
| lncRNA | ENSG00000261765 | 0.94 | 0.39 (0.10-23.41) | 0.41 (0.03-6.98) | 0.5762 |
| lncRNA | UNC13A-AS | 1.07 | 0.05 (0.00-16.06) | 0.09 (0.00-6.98) | 0.5832 |
| lncRNA | PHB-AS | 0.80 | 0.18 (0.02-19.62) | 0.23 (0.04-8.05) | 0.6407 |
| lncRNA | RP11-190A12 | 1.00 | 0.10 (0.01-18.83) | 0.09 (0.00-8.62) | 0.7858 |
| lncRNA | SLC7A9-AS | 0.89 | 0.10 (0.00-38.38) | 0.15 (0.00-16.33) | 0.9224 |
| mRNA | **ITM2B** | 1.23 | 134.3 (20.20-1554.00) | 94.71 (2.00-304.00) | **0.0059** |
| mRNA | EGR1 | 1.36 | 66.67 (8.72-859.70) | 57.39 (4.03-539.30) | 0.0545 |
| mRNA | CXCR4 | 1.10 | 12.74 (1.42-246.10) | 11.42 (0.86-50.08) | 0.1709 |
| mRNA | RGS2 | 0.67 | 7.03 (0.84-84.72) | 10.78 (0.93-171.60) | 0.2486 |

*Wilcoxon test for paired samples (two tailed); FC - fold change; *P*-values in bold are statistically significant.
